# Developing and Validating a Machine Learning Algorithm to Predict the Risk of Incident Opioid Use Disorder Among OneFlorida+ Patients: Prognostic Modeling Study

**DOI:** 10.2196/79482

**Published:** 2026-03-05

**Authors:** Jabed Al Faysal, Weihsuan Lo-Ciganic, Walid F Gellad, Yonghui Wu, Christopher A Harle, Khoa Nguyen, James L Huang, Gerald Cochran, Debbie L Wilson, Stephanie AS Staras, Siegfried OF Schmidt, Eric I Rosenberg, Danielle Nelson, Shunhua Yan, Gary M Reisfield, William M Greene, Courtney Kuza, Md Mahmudul Hasan

**Affiliations:** 1 Department of Pharmaceutical Outcomes & Policy, University of Florida, 1889 Museum Road, Malachowsky Hall, Suite 6300, Gainesville, FL, 32611, United States, 12566946603; 2 Computer Science and Engineering Discipline, Khulna University, Khulna, Bangladesh; 3 Division of General Internal Medicine, School of Medicine, University of Pittsburgh, Pittsburgh, PA, United States; 4 Center for Pharmaceutical Policy and Prescribing, University of Pittsburgh, Pittsburgh, PA, United States; 5 Geriatric Research Education and Clinical Center, North Florida/South Georgia Veterans Health System, Gainesville, FL, United States; 6 Department of Health Outcomes & Biomedical Informatics, College of Medicine, University of Florida, Gainesville, FL, United States; 7 Department of Health Policy and Management, School of Public Health, Indiana University, Indianapolis, IN, United States; 8 Regenstrief Institute, Indianapolis, IN, United States; 9 Department of Pharmacotherapy and Translational Research, College of Pharmacy, University of Florida, Gainesville, FL, United States; 10 Department of Internal Medicine, Division of Epidemiology, University of Utah, Salt Lake City, UT, United States; 11 Department of Community Health and Family Medicine, College of Medicine, University of Florida, Gainesville, FL, United States; 12 Department of Internal Medicine, College of Medicine, University of Florida, Gainesville, FL, United States; 13 Department of Psychiatry, College of Medicine, University of Florida, Gainesville, FL, United States; 14 Department of Information Systems and Operations Management, Warrington College of Business, University of Florida, Gainesville, FL, United States

**Keywords:** opioid use disorder, machine learning, OneFlorida+, risk stratification, external validation

## Abstract

**Background:**

Opioid use disorder (OUD) remains a critical public health crisis in the United States. Despite widespread policy and clinical interventions, early identification of individuals at risk for developing OUD remains challenging due to limitations in traditional screening approaches and a lack of individualized risk stratification methods. Machine learning (ML) methods offer an opportunity to develop timely, high-performing, and explainable predictive models that can enhance OUD prevention strategies in clinical settings.

**Objective:**

This study aims to develop and validate an ML model using electronic health record (EHR) data to predict the 3-month risk of incident OUD among adults initiating opioid therapy and to stratify patients into clinically actionable risk groups.

**Methods:**

This prognostic modeling study used 2017‐2022 OneFlorida+ EHR data to develop and validate ML algorithms predicting 3-month incident OUD risk. We included 182,083 adults (≥18 y) without cancer, overdose, or OUD or hospice history who received ≥1 outpatient, noninjectable opioid prescription. Using 183 predictors measured in sequential 3-month intervals, we developed an elastic net, least absolute shrinkage and selection operator, gradient boosting machine (GBM), and random forest models on randomly split training, testing, and validation sets. Model performance was assessed using C-statistics, predictive values, and number needed to evaluate, with patients stratified into risk deciles for clinical applicability. Model explainability was assessed using Shapley additive explanations, and fairness was evaluated using standard metrics. We externally validated the best-performing model using an independent cohort from the 2018‐2020 UPMC (formerly University of Pittsburgh Medical Center) health system.

**Results:**

In the validation sample (n=60,694), GBM (C-statistics=0.879, 95% CI 0.874‐0.884) and elastic net (C-statistics=0.872, 95% CI 0.867‐0.877) outperformed least absolute shrinkage and selection operator (C-statistics=0.846, 95% CI 0.840‐0.851) and random forest (C-statistics=0.798, 95% CI 0.792‐0.804), with GBM model requiring the fewest predictors (n=75) for predicting 3-month incident OUD. Using the GBM algorithm to predict the subsequent 3-month OUD risk, the top decile subgroup had a positive predictive value of 3.26%, a negative predictive value of 99.8%, and a number needed to evaluate of 31. The top decile (n=6696) captured ~68% of patients with OUD. Shapley additive explanations analysis identified age, number of outpatient visits, history of back and other pain conditions, comorbidity burden, and opioid prescribing patterns as the strongest predictors of incident OUD. Fairness assessment showed an acceptable false negative rate parity across race, age, and sex. In external validation on the UPMC cohort, the GBM model maintained good discrimination (C-statistics=0.756, 95% CI 0.750‐0.762) and effective risk stratification.

**Conclusions:**

An ML algorithm predicting incident OUD derived from OneFlorida+ EHR data performed well in external validation with data using UPMC. The algorithm might be valuable for incident OUD risk prediction and stratification across health systems, with potential to inform early intervention.

## Introduction

The United States continues to face a persistent and evolving opioid epidemic. Opioid use disorder (OUD) and overdose affect millions of Americans, leading to increased morbidity, mortality, and health care costs [1,2] In 2022, more than 6 million individuals experienced OUD, imposing significant societal and economic burdens exceeding US $78 billion annually [1,3,4]. Opioid-related deaths increased tenfold from 1999 to 2022 (>82,000 in 2022) [5-8]. Prescription opioids were responsible for approximately 280,000 overdose deaths over the same period [9]. The prevalence of OUD continues to rise despite efforts to curb opioid misuse [10,11]. In response, health systems, payers, and policymakers have implemented various interventions aimed at reducing unsafe prescribing and patient risk [12].

Early identification of OUD can prevent severe addiction, high-risk behaviors, overdose, and death, while enabling timely access to evidence-based treatments such as buprenorphine or referral to recovery services. Existing methods for identifying high-risk opioid users, such as high-dose opioid prescribing thresholds and use of multiple pharmacies, are often based on simple or single criteria [13,14]. These rule-based strategies leave many high-risk individuals undetected and may misclassify others, resulting in unintended consequences like delayed care, unnecessary monitoring, or stigma. For example, a study found that nearly 43% of individuals who developed OUD were missed by such approaches [15]. Furthermore, traditional statistical models emphasize population-level risk factors and assume linear relationships between predictors and outcomes, limiting their ability to capture complex interactions among patient demographics, comorbidities, health care use, and prescriber patterns. Although tools such as the Screener and Opioid Assessment for Patients with Pain-Revised and the Opioid Risk Tool are commonly used in clinical settings, studies report inconsistent estimates of test accuracy and no evidence that they reduce OUD or overdose [16-18]. The 2022 Centers for Disease Control and Prevention Clinical Practice Guideline similarly concluded that current opioid risk screening tools lack proven clinical use. Proprietary platforms such as NarxCare leverage prescription and health record data but lack transparency, public validation, and demonstrated generalizability [19]. These limitations underscore the need for more robust, transparent, and generalizable models to support real-time clinical decision-making across diverse populations. Studies have also highlighted these shortcomings and called for more advanced, data-driven models to improve identification of individuals at risk (or low risk) of OUD [20-24].

Machine learning (ML) offers a promising alternative to traditional statistical methods in improving risk prediction. ML models can analyze large health care data to identify complex nonlinear patterns in patient data. Recent studies have demonstrated the potential of ML to predict opioid-related outcomes, including OUD and overdose, with higher accuracy than that of traditional methods [25-28]. Our previous work showed that ML approaches can improve risk prediction and stratification for incident OUD and subsequent overdose in Medicare and Medicaid beneficiaries. For example, we developed ML models to predict incident OUD among Medicare beneficiaries, achieving high discrimination (C-statistic >0.86) and effectively stratifying patients into clinically meaningful risk subgroups (eg, low, moderate, and high risk for developing OUD) [29]. However, many existing models are developed using Medicare or Medicaid data, which primarily include older adults or low-income populations.

In this study, we extend our work to develop and validate an ML algorithm to predict incident OUD among patients who received care from institutions within the OneFlorida+ network—a large, diverse patient population across Florida, Georgia, Alabama, and Arkansas, including individuals with commercial insurance, Medicaid, or Medicare, and those with no insurance [30]. This study advances our prior Medicare-based work in several important ways. First, although claims-based models can be incorporated into some clinical workflows, electronic health records (EHRs) often lack the full use and medication detail available in claims and may vary in data quality. As such, adapting the model for EHR-based use required methodological refinement to support real-time, point-of-care integration. Second, the broader and more heterogeneous cohort enhances the model’s generalizability beyond the older, fee-for-service Medicare population used previously. Third, we externally validated the model using an independent cohort from the UPMC health system, assessing cross-system generalizability and transportability—an important step toward real-world implementation. Fourth, we incorporated fairness and bias analyses across demographic subgroups to address equity considerations often overlooked in prior OUD risk prediction studies. Finally, we articulate a translational vision for integrating this model into clinical workflows. By organizing predictors to enable risk-based stratification, the model is designed to function as an EHR-embedded decision support tool in primary care settings, alerting providers (eg, physicians) when a patient is flagged as high-risk to prompt timely, preventive actions. We emphasize model interpretability to ensure that our findings can be seamlessly integrated into the clinical environment.

## Methods

### Study Design and Data Source

This is a prognostic modeling study with a retrospective cohort design. We used the TRIPOD+AI (Transparent Reporting of a Multivariable Prediction Model for Individual Prognosis or Diagnosis + Artificial Intelligence) guidelines for reporting our work (Checklist 1) [31]. This study used data from OneFlorida+, a secure and centralized repository of patient‐level data from both health system partners and insurers, and managed by the OneFlorida Clinical Research Consortium [32]. Currently, OneFlorida+ includes EHRs and claims data from 26 million individuals across Florida, Georgia, Alabama, and Arkansas, covering a wide range of populations and health care settings [30,33]. All data sources are mapped to the Patient‐Centered Outcomes Research network common data model (version 7.0) to ensure standardization of data elements across sources. Major data elements in the common data model include demographics, enrollment, encounters, diagnoses, procedures, dispensed medications, and deaths. Additionally, while OneFlorida data includes Florida Medicaid recipients with claims data only, our study cohort requires having at least 1 EHR encounter. The study population included adult patients (aged ≥18 y) who had at least 1 opioid prescription filled between 2017 and 2022 (Figure S1 in Multimedia Appendix 1). The index date was defined as the date of a patient’s first opioid prescription between October 1, 2017, and September 30, 2022. To construct a cohort appropriate for assessing incident OUD risk, we excluded patients who (1) had cancer diagnoses (Table S1 in Multimedia Appendix 2), (2) received hospice care, (3) had a diagnosis of OUD, opioid overdose, other substance use disorders, drug abuse, or received methadone or buprenorphine for OUD before initiating opioids, or (4) did not have at least a 3-month observation window following the first opioid prescription to allow for predictor measurement. We also excluded patients with a diagnosis of other substance use disorders to minimize confounding, as some physicians may have used this diagnosis for patients with co-occurring OUD and other substance use conditions. Once eligible, patients remained in the cohort until they experienced an outcome of interest (ie, incident of OUD diagnosis) or were censored due to death, regardless of continued opioid use (Figure S2 in Multimedia Appendix 1).

For external validation, we applied the algorithm to 2018‐2020 EHR data from the UPMC health system in Pennsylvania, a region with demographic and opioid prescribing characteristics distinct from the OneFlorida+ network. Cohort construction and predictor generation followed the same procedures used for the OneFlorida+ dataset.

### Outcome Variable

Similar to other claims-based analyses [29,34-36], our primary outcome was incident OUD (Table S2 in Multimedia Appendix 2), defined as the first recorded diagnosis of OUD from all settings in each 3-month window after the index prescription date (Figure S2 in Multimedia Appendix 1). The *ICD-10* (*International Statistical Classification of Diseases and Related Health Problems 10th Revision*) codes for incident OUD diagnosis include F11.1X and F11.2X but exclude F11.11 (opioid-related disorders in remission) and F11.21 (opioid dependence in remission).

### Predictor Candidates

We identified 183 candidate predictors of OUD from the prior literature (Table S3 in Multimedia Appendix 2) [37-48]. These predictors included sociodemographic factors, patient health status, and patterns of opioid use and other nonopioid prescriptions measured at baseline (during the 3 month period before the first opioid fill) and in 3-month windows after initiating prescription opioids. This 3-month window was selected for both clinical and operational relevance. Evidence shows that early indicators of problematic opioid use (eg, dose escalation, early refills, or polypharmacy) typically emerge within the first 3 months of therapy [49-52]. Pharmacoepidemiologic studies demonstrate that transitions to sustained or high-risk opioid use most commonly occur within 3 months after treatment initiation [53,54]. This timeframe also aligns with the quarterly monitoring cycles used by many prescription drug monitoring programs and health plans [14,38]. We updated the predictors measured in each 3-month period to account for changes over time for predicting incident OUD risks in each subsequent period (Figure S2 in Multimedia Appendix 1). This time-updating approach mimics active surveillance that a health system might conduct in real time [55] to provide clinicians with timely, actionable opportunities (eg, closer follow-up, patient counseling, or medication adjustments) before the progression to OUD. We examined missingness across candidate predictors and applied prespecified imputation rules before model training. We used simple imputation techniques where missing values for all categorical predictors (including race, ethnicity, and provider sex) were imputed using the modal category. Continuous predictors (eg, use counts, comorbidity scores, and medication counts) with missing values were imputed using the median calculated from the full analytic dataset. Binary and count predictors representing the presence or frequency of diagnoses, procedures, or medications were imputed as zero when missing, consistent with the interpretation that the absence of a code reflects no recorded event during the baseline window.

### ML Approaches and Prediction Performance Evaluation

Our machine learning analysis using OneFlorida+ data had two objectives: (1) developing a prediction model to generate individuals’ incident OUD risk scores and (2) stratifying individuals into subgroups based on similar OUD risk levels. We developed and tested 4 ML algorithms to predict incident OUD: elastic net (EN), least absolute shrinkage and selection operator (LASSO), gradient boosting machine (GBM), random forest (RF). These algorithms have consistently demonstrated strong predictive performance in prior studies [12,46,56,57]. Details of each algorithm are provided in Multimedia Appendix 3. We randomly divided the cohort into 3 subsets: a training sample for algorithm development, a testing sample for algorithm refinement, and a validation sample for evaluating predictive performance. This split was conducted strictly by patient ID, such that all 3-month episodes from a given patient were assigned to a single dataset (training, testing, or validation), and no patient contributed episodes to more than one set, thereby avoiding information leakage across datasets. Because patients could contribute multiple 3-month episodes, episode-level observations within patients were correlated by design. Models were trained and evaluated at the episode level to reflect the intended real-world use case of repeated, longitudinal risk assessment over time and to maximize learning from rare outcomes. Tree-based ensemble methods such as GBMs are generally robust to correlated observations because they do not rely on independence assumptions and learn predictive structure through recursive partitioning rather than parametric estimation. To evaluate the potential impact of within-patient correlation on model performance estimates, we conducted sensitivity analyses using patient-level random subsets. We used one 3-month period with predictor measurements to forecast risk in the subsequent 3 months within the validation set.

To evaluate discrimination performance, we assessed whether individuals predicted to be at high risk had higher OUD incidence than those predicted to be at low risk. We compared C-statistics across different methods in the validation sample using the DeLong test [58]. A C-statistic between 0.7 and 0.8 indicates good discrimination, while values above 0.8 indicate very good discrimination. Precision-recall curves were also examined [42]. Given that OUD events are rare outcomes and C-statistics do not incorporate information about outcome incidence, we reported other metrics, including sensitivity, specificity, positive predictive value (PPV), negative predictive value (NPV), number needed to evaluate (NNE) to identify 1 OUD episode, and estimated rate of alerts, to thoroughly assess the algorithms’ predictive ability (Figure S3 in Multimedia Appendix 1) [43,59].

There is no universally applicable prediction probability threshold, so we evaluated performance across multiple sensitivity and specificity levels (eg, selecting 90% sensitivity). To determine an optimized threshold that balances sensitivity and specificity, we applied the Youden index in the training sample [60]. In the validation sample, patients were stratified into risk subgroups based on deciles of predicted incident OUD probability. The highest decile was further divided into 3 strata: the top first percentile, the second to fifth percentiles, and the sixth to tenth percentiles. This approach allowed for a more detailed assessment of individuals at the highest risk of developing OUD.

We conducted additional analyses to enhance clinical use. The primary goal of our ML algorithm was to generate risk scores for incident OUD. For comprehensive model explanation and further enhancement of model interpretability, we conducted a Shapley additive explanations (SHAP) analysis [61]. SHAP quantifies the contribution of each predictor to the model’s output while accounting for interactions with other features. The SHAP plots indicate whether the mentioned features have a positive correlation (red color) or a negative correlation (blue color) with OUD. To evaluate bias across demographic factors (eg, race, age, and sex), we examined risk score distributions and false positive rates (FPR) and false negative rates (FNR) in the best performing model. We defined significant disparity as a parity ratio between subgroups falling outside the range of 0.80 to 1.25, consistent with the standards used in algorithmic fairness audits [62].

Additionally, we evaluated model calibration to assess the reliability of predicted risk scores. Given the potential for miscalibration in rare-outcome ML models, we applied isotonic regression to recalibrate predicted risk scores [63]. The overall accuracy of probabilistic predictions was assessed using the Brier score metric [64]. To determine clinical use beyond discrimination, we performed decision curve analysis. We calculated the net benefit [65] across a range of clinically relevant risk thresholds, comparing the recalibrated model against default strategies of “treat all” and “treat none”. This approach quantifies the trade-off between the benefit of identifying true OUD cases and the potential harm of false-positive alerts.

### Statistical Analysis

All analyses were performed using SAS 9.4 (SAS Institute Inc) and Python 3.6 (Python Software Foundation). Continuous variables were summarized using mean (SD) values, while categorical variables were summarized using frequencies and percentages. We compared patients’ characteristics in training, testing, and validation datasets using 2-tailed Student *t* tests, chi-square tests, and ANOVA, or corresponding nonparametric tests.

### Ethical Considerations

This study was reviewed and approved by the University of Florida (UF) Institutional Review Board (IRB202101897). The research involved secondary analysis of limited electronic health record data from the OneFlorida+ Data Trust. In accordance with federal regulations (45 CFR 46.104[d] [4]) and institutional policies, the requirement for informed consent was waived because the study involved no direct participant contact and posed minimal risk to individuals. All analyses were conducted within the secure UF server environment; no identifiable private information was accessed or transmitted outside the system. The study adhered to institutional data-use agreements and complied with all relevant guidelines for human subjects research and data privacy. The external validation using UPMC data was reviewed by the University of Pittsburgh Human Research Protection Office and determined not to constitute human subjects research, as only deidentified data were used. Therefore, institutional review board review and informed consent were not required.

## Results

### Patient Characteristics

OneFlorida+ patients in the training (n=60,694), testing (n=60,695), and validation (n=60,694) samples had similar characteristics and outcome distributions (mean age 53.02, SD 7.60 y; 111,576/182,083, 61.28% female; 125,208/182,083, 68.76% White; 144,316/182,083, 79.26% non-Hispanic; 4328/182,083, 2.38% had incident OUD; Table 1). The external validation cohort from the UPMC included 129,215 patients (mean age 58.91, SD 16.75 y; 78,574/129,215, 60.81% female; 115,907/129,215, 89.7% White; 123,083/129,215, 95.25% non-Hispanic; 2537/129,215, 1.96% had incident OUD). While sex distributions were similar between the 2 health systems, the UPMC cohort was older and less diverse, with more White and non-Hispanic patients.

**Table 1.** Selected characteristics of OneFlorida+ and UPMC^a^ health system patients.

Characteristics	2017‐22 OneFlorida+ data			2018‐20 UPMC^a^ data
	Training (n=60,694)	Testing (n=60,695)	Internal validation (n=60,694)	External validation (n=129,215)
Development of incident opioid use disorder, n (%)	1443 (2.38)	1433 (2.36)	1452 (2.39)	2537 (1.96)
Age (years), mean (SD)	53.11 (17.57)	52.98 (17.64)	52.98 (17.57)	58.91 (16.75)
Age group (years), n (%)				
18‐34	10,200 (16.81)	10,286 (16.95)	10,285 (16.95)	13,852 (10.73)
35‐50	15,326 (25.25)	15,501 (25.54)	15,477 (25.5)	23,931 (18.52)
51‐64	19,121 (31.5)	19,067 (31.41)	19,096 (31.46)	44,047 (34.10)
≥65	16,047 (26.44)	15,841 (26.1)	15,836 (26.09)	47,385 (36.65)
Sex, n (%)				
Male	23,534 (38.77)	23,489 (38.70)	23,481 (38.69)	50,641 (39.19)
Female	37,160 (61.23)	37,206 (61.30)	37,210 (61.31)	78,574 (60.81)
Race, n (%)				
White	41,737 (68.77)	41,751 (68.79)	41,720 (68.74)	115,907 (89.70)
Black	13,403 (22.08)	13,458 (22.17)	13,548 (22.32)	10,833 (8.38)
Other or unknown	5554 (9.15)	5486 (9.04)	5426 (8.94)	2475 (1.92)
Ethnicity, n (%)				
Non-Hispanic	47,951 (79)	48,242 (79.48)	48,123 (79.29)	123,083 (95.25)
Hispanic	10,756 (17.72)	10,455 (17.23)	10,540 (17.37)	1013 (0.78)
Other or unknown	1987 (3.27)	1998 (3.29)	2031 (3.35)	5123 (3.96)

a UPMC: Formerly known as University of Pittsburgh Medical Center.

### Prediction Performance Across ML Methods

**Figure 1.**
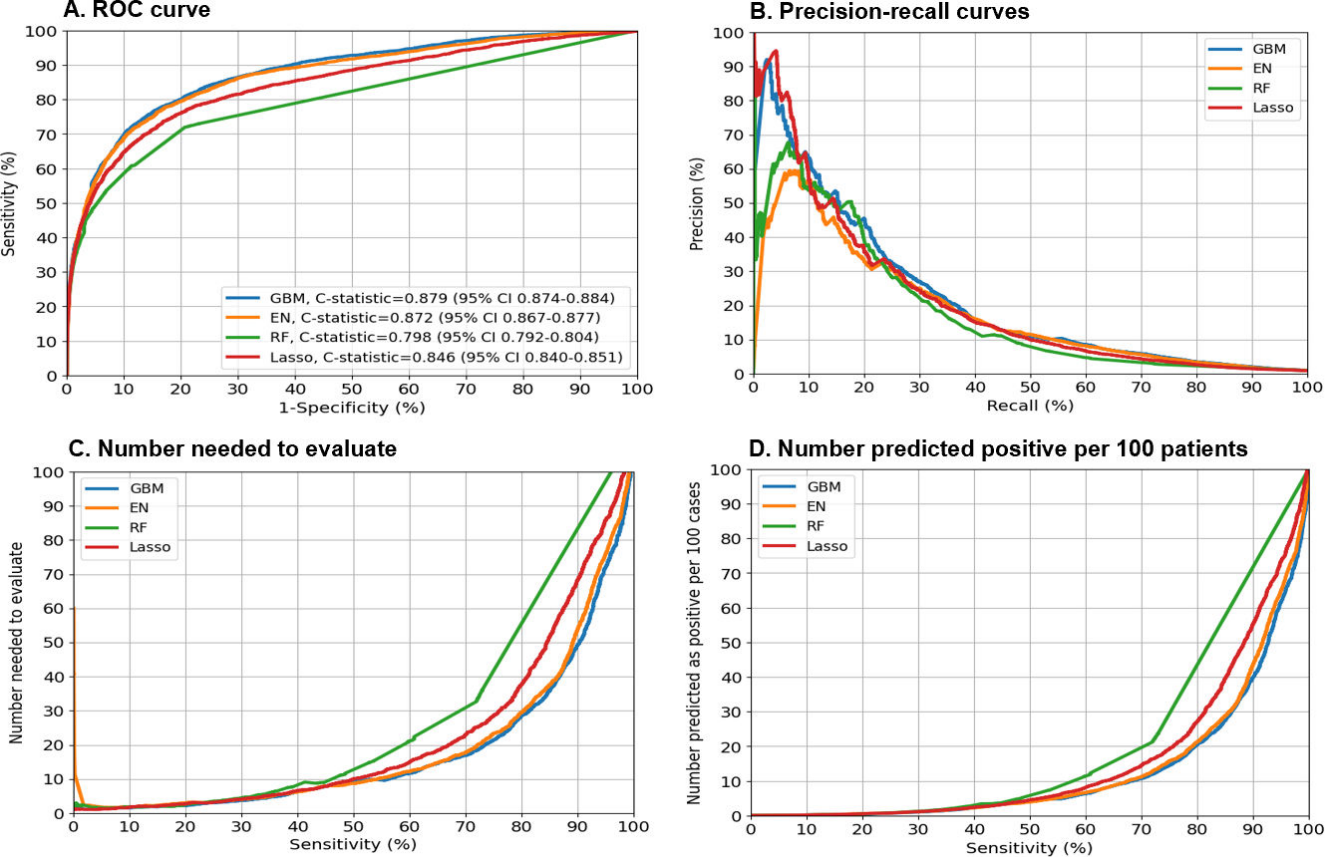
Performance matrix across machine learning models for predicting incident opioid use disorder in OneFlorida+ patients. This figure shows 4 prediction performance matrices in the validation sample. (A) The areas under receiver operating characteristic curves (or C-statistics). (B) The precision-recall curves (precision=positive predictive value and recall=sensitivity): precision-recall curves that are closer to the upper right corner or are above another method have improved performance. (C) The number needed to evaluate by different cutoffs of sensitivity. (D) Alerts per 100 patients by different cutoffs of sensitivity. AUC: area under the curve; EN: elastic net; GBM: gradient boosting machine; LASSO: least absolute shrinkage and selection operator; PPV: positive predictive value; RF: random forest; ROC: receiver operating characteristics.

Figure 1 summarizes the 4 prediction performance measures of each model in the internal validation sample. GBM (C-statistics=0.879, 95% CI 0.874‐0.884) and EN (C-statistics=0.872, 95% CI 0.867‐0.877) models outperformed the LASSO (C-statistics=0.846, 95% CI 0.840‐0.851) and RF (C-statistics=0.798, 95% CI 0.792‐0.804) models, with the GBM model requiring the fewest predictors (n=75) for predicting 3-month incident OUD (*P*<.001; Figure 1A). The GBM and EN models had similar prediction performance, and the GBM model had the best precision-recall performance (Figure 1B). Sensitivity analyses using patient-level data yielded similar results (Figure S4 in Multimedia Appendix 1). To evaluate model robustness across key clinical subgroups, we stratified the GBM model’s performance by age, sex, and ethnicity. The model demonstrated consistent discriminative ability across populations, with C-statistics ranging from 0.833 to 0.909 across age groups (Figure S5 in Multimedia Appendix 1), 0.865 to 0.887 between sexes (Figure S6 in Multimedia Appendix 1), and 0.864 to 0.914 by ethnicity (Figure S7 in Multimedia Appendix 1).

Table S4 in Multimedia Appendix 2 shows the performance measures for predicting incident OUD across different levels (90%‐100%) of sensitivity and specificity for GBM and EN. When set at the optimized sensitivity and specificity as measured by the Youden index, the GBM model had a sensitivity of 76.59%, specificity of 84.75%, PPV of 4.36%, NPV of 99.75%, and NNE of 23; and the EN model had a sensitivity of 77.65%, specificity of 82.74%, PPV of 3.93%, NPV of 99.76%, and NNE of 25 (Figures 1C and 1D; Table S4 in Multimedia Appendix 2).

### Risk Stratification, Bias and Fairness Analysis, and Model Explainability

**Figure 2.**
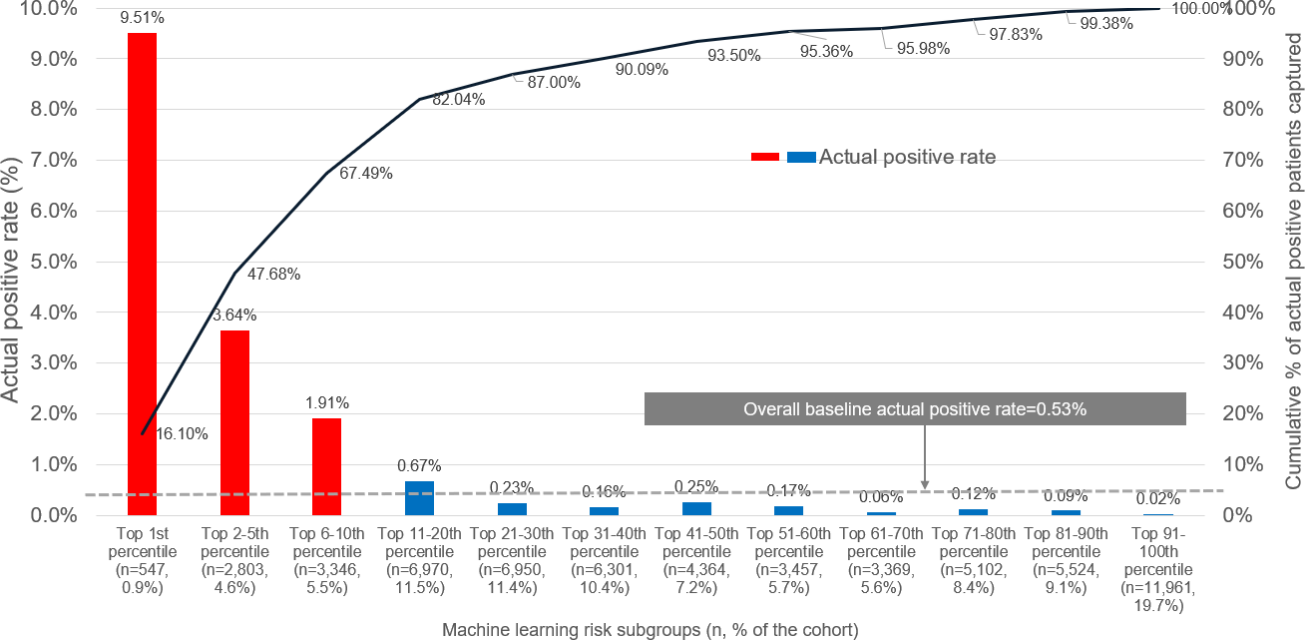
Incident opioid use disorder was identified by the gradient boosting machine’s decile risk subgroup in the validation sample. Based on the individual’s predicted probability of an opioid use disorder event, we classified patients in the internal validation sample into decile risk subgroups, with the highest decile further split into 3 additional strata based on the top first, second to fifth, and sixth to tenth percentiles to allow closer examination of patients at the highest risk of developing opioid use disorder.

Figure 2 represents the actual OUD rate for individuals in each decile subgroup using GBM. The high-risk subgroup (with risk scores in the top decile; 11% [n=6696] of the validation cohort) had a PPV of 3.26%, a NPV of 99.8%, and NNE of 31. In a hypothetical clinical application involving 1000 patients, applying the top decile threshold would generate alerts for approximately 100 individuals. Based on the observed PPV at this threshold, approximately 4 of the 100 flagged patients would be expected to develop OUD, demonstrating the model’s ability to meaningfully concentrate risk among a small subgroup. Conversely, the high NPV (99.8%) indicates that very few incident OUD cases would arise among the 900 unflagged patients, consistent with the model’s low false negative rate. Taken together, these estimates illustrate how the model could support prioritization of patients most likely to benefit from closer monitoring or preventive intervention.

Among all 323 individuals with an incident OUD, 265 (82.04%) occurred in the top two decile subgroups (Decile 1=67.5 % and Decile 2=14.6%). Those in the first decile subgroup had at least a 14-fold higher OUD rate compared to the lower-risk groups (eg, observed OUD rate: Decile 1=9.51%, Decile 2=0.67%, Decile 10=0.02%). The third through tenth decile subgroups had minimal OUD incidence rate (2 to 16 per 10,000).

In our evaluation of racial fairness, we found that among the true positive cases identified in the top decile risk subgroup by the GBM model, 78.44% (171/218) were White individuals and 18.8% (41/218) were Black individuals. In the top first percentile, the distribution was 67.3% (35/52) White individuals and 25% (13/52) Black individuals. The detailed racial distribution of true positives across all risk subgroups is provided in Table S5 in Multimedia Appendix 2. We also compared FNR and FPR by race in the GBM model with and without race as a predictor. FNRs were consistently higher for Black individuals compared to White individuals across most percentiles, while FPR remained similar across racial groups (Figure S8 and Figure S9 in Multimedia Appendix 1). We compared the FNR and FPR between racial groups at the 90th percentile and found that the FNR parity ratio (1.23) and FPR parity ratio (1.03) were within the accepted threshold (0.8‐1.25) [62], suggesting equitable model performance across racial groups when race was included as a predictor. We also assessed fairness across age and sex. The true positive cases in the top decile were concentrated among the 35‐ to 50-year age group (82/218, 37.61%) and females (146/218, 66.97%). The detailed distribution of true positives by age and sex across all risk subgroups is provided in Tables S6 and S7 in Multimedia Appendix 2. FNRs were slightly higher for older individuals (≥65 y) across most percentiles compared to the young adults (18‐34 y), while FNRs for males and females were closely aligned. FPRs remained consistent across both age groups and sex (Figures S10 and S11 in Multimedia Appendix 1). Parity ratios for sex (FNR=0.91, FPR=1.18) and age-based FPR (0.91; comparing 18‐34 vs ≥65 y) remained within the acceptable range (0.8‐1.25), whereas the age-based FNR parity ratio (0.74) was below the threshold.

**Figure 3.**
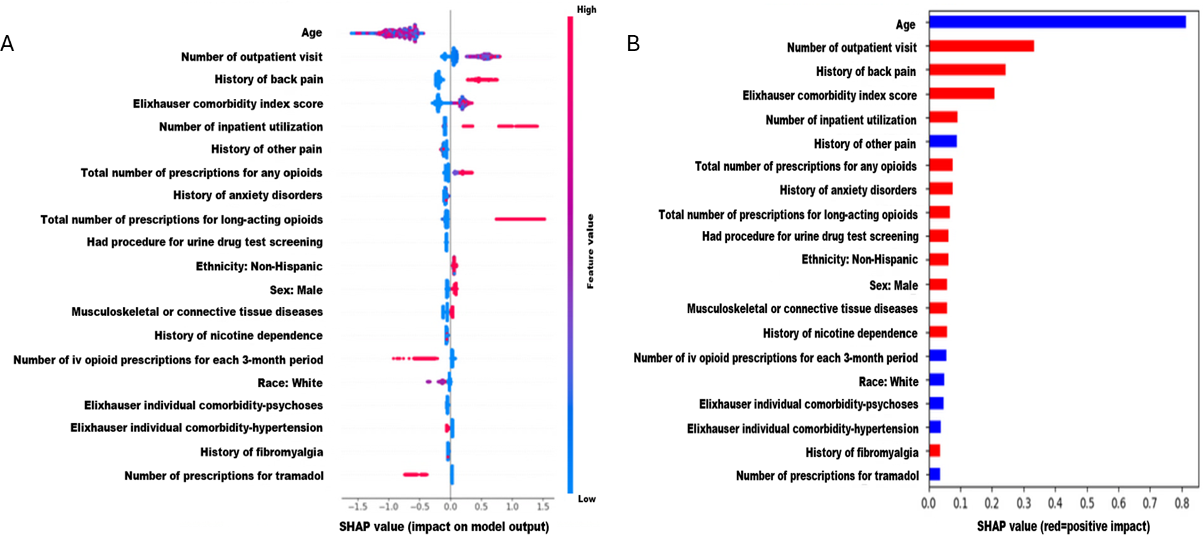
Variable importance plots using Shapley additive explanations values from the gradient boosting machine model. (A) Shapley additive explanations value computed from individual features’ values and their impact (both positive and negative) on incident opioid use disorder. (B) Average Shapley additive explanations values of features showing average impact on and correlation with incident opioid use disorder. SHAP: Shapley additive explanations.

Figure 3 presents variable importance plots using SHAP values derived from the GBM model. The plots on the right-hand side present the average impact of various features on incident OUD, listed in descending order of their significance. Variables such as age and history of other pain for each 3-month period were negatively correlated with incident OUD, while factors having a positive correlation with incident OUD included the number of outpatient visits, history of back pain, and Elixhauser comorbidity index score.

### Calibration and Decision Curve Analysis

Calibration assessment showed that the GBM model initially overestimated risk (Brier score=0.09), a known phenomenon in rare-event prediction. After isotonic recalibration, model reliability improved significantly, with a calibration slope of 0.98 (ideal=1), an intercept of –0.03 (ideal=0), and a Brier score of 0.01 (lower is better; Figure S12 in Multimedia Appendix 1). Decision-curve analysis showed that a “treat-all” strategy (intervene on everyone) yields negative net benefit at risk thresholds above 0.5% (Figure S13 in Multimedia Appendix 1), whereas the GBM model maintained positive net benefit across a broader range of clinically relevant thresholds. For example, at the top decile cutoff (probability threshold=0.009), the model achieved a net benefit of 0.0026. This corresponds to identifying approximately 2.6 additional true-positive OUD cases per 1000 patients (after accounting for false-positive alerts) compared with a strategy of no intervention. Relative to the OUD outcome incidence (0.53% in the prediction window), this net benefit reflects ~50% of the maximum achievable use for a perfect classifier in this setting, indicating substantial clinical value despite the low-prevalence context.

### External Validation Performance

When applied to the UPMC cohort, the best-performing GBM model achieved a C-statistic of 0.756 (95% CI 0.750‐0.762; Figure S14 in Multimedia Appendix 1). The high-risk decile subgroup demonstrated a PPV of 2.52%, an NPV of 99.8%, and NNE of 40 (Figure S15 in Multimedia Appendix 1). The top first percentile had an OUD incidence rate 14-fold higher than the overall baseline (8.15% vs 0.58%). Fairness analyses in the UPMC cohort revealed variations in FNR and FPR across age groups (Figure S16 in Multimedia Appendix 1), while error rates remained similar across race (Figure S17 in Multimedia Appendix 1) and between males and females (Figure S18 in Multimedia Appendix 1). For example, using the 90th percentile risk score as the elevated risk cutoff, younger adults (18‐34 y) had a lower FNR (fewer missed cases) compared to older adults (≥65 y).

## Discussion

### Principal Findings

This study expanded our previous work using ML approaches to improve the accuracy of predicting incident OUD in the subsequent 3 months of prescription opioid initiation among fee-for-service Medicare beneficiaries and broaden the applicability of these models across a diverse population in the OneFlorida+ network [29]. Our GBM and EN models demonstrated strong predictive performance, achieving a high C-statistic (>0.87) and outperforming LASSO and RF. Our best-performing GBM model offers several advantages, including its ability to eliminate the need for a separate feature selection process and its flexibility in hyperparameter tuning to capture complex interactions between predictors and outcomes. We acknowledge that this flexibility in model tuning can be computationally intensive and time-consuming. The algorithm effectively stratified the population into distinct risk groups based on predicted risk scores. Notably, approximately 80% of the cohort had minimal OUD risk, while the highest-risk decile alone accounted for ~68% (218/323) of all individuals who developed OUD. Given the low incidence of OUD within a 3-month period, the PPV was expectedly low. However, in the context of OUD prevention, a lower PPV may be ethically and clinically acceptable when associated interventions are supportive (eg, closer follow-up, motivational interviewing, review of opioid therapy, or other high-risk concurrent medication use) rather than punitive actions (eg, refusal to prescribe or abrupt discontinuation). For these low-risk, supportive interventions, the potential harm of a false positive (providing additional support to a patient who would not have developed OUD) is minimal compared with the harm of missing a true high-risk case (false negative). It is therefore important to frame the model as augmenting, not replacing, clinician judgment to ensure that low PPV does not lead to overreaction to false positives or unintended consequences.

A key strength of this study is the successful external validation of our algorithm using an independent UPMC cohort. Our model maintained good performance (C-statistic: 0.76) without retraining, demonstrating transportability across two distinct health care systems. This replication supports the stability of the key predictors across settings and indicates that the model can effectively identify high-risk individuals in diverse clinical environments.

Prior studies, targeting different aspects of OUD risk, varied in their prediction windows and data sources, including 6-month OUD risk based on private insurance claims [66]; 12-month risk of aberrant opioid use behaviors following an initial pain clinic visit [67]; and 12-month OUD risk using private insurance claims [38,68] or pharmacy benefit manager claims data [36]. Other models focused on longer-term risk, such as 2-year problematic opioid use documented in EHR from primary care [69] and 5-year OUD risk using electronic medical record from a medical center [34] or Rhode Island Medicaid data [35]. Despite their contributions, these models had key limitations. Most measured predictors are only at baseline, lacking temporal updates over time. Several relied on case-control designs, which may not generalize well to population-level OUD incidence rates. Additionally, even in non–case-control designs, the highest reported C-statistic was 0.85, indicating room for improvement in predictive performance [35,36,67,69]. Expanding on our prior Medicare claims-based study, this work explored whether predictive performance can be refined using EHR data. Our study addressed the abovementioned limitations by using a population-based cohort and a more immediate prediction window, estimating OUD risk within the next 3 months rather than over a year or longer. This short-term predictive approach, combined with innovative risk stratification and enhanced clinical interpretability, made our method highly applicable for timely intervention.

### Limitations

Although the findings of the study are promising, some limitations need to be acknowledged. First, incident OUD was identified solely through *ICD-10* diagnosis codes, which may undercapture true OUD cases due to known under-coding, variability in diagnostic practices, and reluctance to document substance use disorders in clinical settings. As a result, some individuals with OUD may have been misclassified as noncases. Although prior work has similarly relied on diagnosis-based definitions [29,34-36], broader definitions incorporating buprenorphine treatment or combinations of high-risk prescribing behavior indicators may improve case ascertainment. Second, unmeasured predictors, such as socioeconomic determinants and illicit opioid use, could influence risk trajectories. Third, while the study uniquely assessed OUD risk within 3 months of opioid initiation, its reliance on older EHR data may limit real-time applicability due to potential delays in data availability and processing.

### Clinical Utility

From a clinical use perspective, the model’s ability to stratify patients by risk supports early intervention and more efficient resource allocation in primary care settings. Our analysis confirms that the model’s risk estimates are not only accurate in distinguishing higher- from lower-risk patients but also well calibrated after post hoc adjustment. Decision-curve analysis further demonstrated that the model offers meaningful clinical utility across thresholds where default “treat-all” strategies offer little value in the context of a low outcome incidence. At the top decile threshold, the model achieved a positive net benefit equivalent to identifying approximately 2.6 additional true OUD cases per 1000 patients—representing about 50% of the maximum achievable use given the cohort’s baseline incidence. These findings align with established decision-analytic frameworks [70] and highlight the model’s potential to effectively prioritize individuals most likely to benefit from preventive intervention. Integrating such models into clinical decision support (CDS) tools could enable providers to proactively flag and manage patients at elevated risk for OUD before symptoms escalate. However, ethical considerations must also be addressed, including the potential for unintentionally reinforcing existing health disparities. Although the overall FNR and FPR parity ratio was within acceptable bounds, Black patients exhibited higher false negative rates across percentiles, suggesting that true OUD cases may be underidentified in this group. To address this disparity, we outline potential mitigation strategies for future model refinement. These include group-specific threshold adjustment to harmonize error rates, reweighting or cost-sensitive learning to shift model attention toward underidentified subgroups, and postprocessing approaches such as calibration-by-group or equalized-odds adjustment. Importantly, any fairness intervention should balance reducing disparities with avoiding unintended undertreatment or new forms of inequity. These considerations underscore the need for iterative fairness evaluation as part of a responsible deployment framework.

### Clinical Implementation and Future Directions

To support real-world deployment, our translational roadmap includes a structured, multiphase implementation strategy grounded in principles of human-centered design and clinical workflow alignment. In the retrospective phase, we will operationalize the OUD risk model within the UF Health Integrated Data Repository, a comprehensive enterprise clinical data warehouse. This step will enable validation using real-time patient data and ensure backend compatibility with the Epic EHR system. The model will generate individual-level risk scores for patients with recent opioid prescriptions, using secure internal infrastructure and consistent EHR data formatting. In the next phase, we will begin with a feasibility test and pilot integration of the model into the EHR system (Epic) within UF Health, gathering real-world feedback from clinicians before scaling up. Notably, UF Health’s Epic EHR infrastructure has already implemented a system-wide approach for an artificial intelligence–driven CDS tool that predicts patients with a high risk of opioid overdose in primary care workflows [71,72].

In the near term, we plan to deploy the OUD risk prediction model through EHR-based CDS tools that seamlessly integrate into routine care. The model’s risk score will trigger automated alerts during opioid prescribing encounters, prompting timely interventions for those identified as high risk. For example, in a primary care clinic with approximately 1000 patients initiating opioid therapy annually, applying a top decile (10%) risk threshold would flag approximately 100 individuals as high risk, of whom ~4 would be expected to develop OUD within 3 months. Although the PPV remains low due to the rarity of incident OUD, the alert volume is manageable in routine practice, particularly when recommended interventions are supportive and low-intensity (eg, increased follow-up, medication review, and motivational interviewing). The remaining ~90% low-risk patients would have an extremely high NPV, allowing providers to continue appropriate pain management for those individuals without unnecessary alarm. To further address alert fatigue, we propose specific operational strategies such as (1) embedding alerts within existing opioid or pain management workflows (eg, triggering only when signing a new medication order) to minimize disruption, (2) using tiered alerting triggered by repeated high-risk predictions, and (3) combining risk scores with other clinically meaningful indicators (eg, concurrent benzodiazepine prescribing) to enhance alert specificity. To minimize potential harms such as stigma and inappropriate opioid restriction, alerts should be framed using universal-precaution language that encourages supportive monitoring rather than implying misuse.

Although our findings indicate that the model has characteristics that could support future integration into clinical workflows, real-world feasibility and impact have not yet been evaluated. Additional implementation studies, including usability testing, workflow integration, and prospective evaluation, are needed before broad deployment in routine practice. While our separate pilot trial, Developing and Evaluating a Machine Learning Opioid Prediction & Risk-Stratification E-Platform, is currently assessing the feasibility of integrating an EHR-based overdose alert into clinical workflows within UF Health primary care clinics [71,72], analogous testing would be required for the model developed in this study.

### Conclusions

This study demonstrates that an ML algorithm derived from the OneFlorida+ network can accurately predict incident OUD and effectively stratify risk across diverse populations and health systems, as evidenced by successful external validation in UPMC. The next step is to prospectively validate this algorithm within the UF Health clinical workflow to leverage existing decision support infrastructure and facilitate early intervention. Future research should expand the model to incorporate unstructured clinical notes, prescription drug monitoring program data, and social drivers of health (eg, housing instability, employment status, and incarceration history) to further improve prediction accuracy and equity.

## Supplementary material

10.2196/79482 Multimedia Appendix 1 Study design, model performance (internal and external validation), calibration and decision curves, and risk stratification.

10.2196/79482 Multimedia Appendix 2 Diagnosis codes, predictor definitions, and model performance.

10.2196/79482 Multimedia Appendix 3 Methods for machine learning model development and evaluation.

10.2196/79482 Checklist 1 TRIPOD+AI checklist.

## Abbreviations

CDS: clinical decision support
EHR: electronic health record
EN: elastic net
FNR: false negative rate
FPR: false positive rate
GBM: gradient boosting machine
*ICD-10*: *International Statistical Classification of Diseases and Related Health Problems 10th Revision*
LASSO: least absolute shrinkage and selection operator
ML: machine learning
NNE: number needed to evaluate
NPV: negative predictive value
OUD: opioid use disorder
PPV: positive predictive value
RF: random forest
SHAP: Shapley additive explanations
TRIPOD+AI: Transparent Reporting of a Multivariable Prediction Model for Individual Prognosis or Diagnosis+Artificial Intelligence
UF: University of Florida
UPMC: University of Pittsburgh Medical Center
