# Supplementary material for: Developing and Validating a Machine Learning Algorithm to Predict the Risk of Incident Opioid Use Disorder Among OneFlorida+ Patients: Prognostic Modeling Study
Source: J Med Internet Res. 2026 Mar 5;28:e79482. doi: 10.2196/79482 (PMC12978897; doi:10.2196/79482)

**Figure S1.**


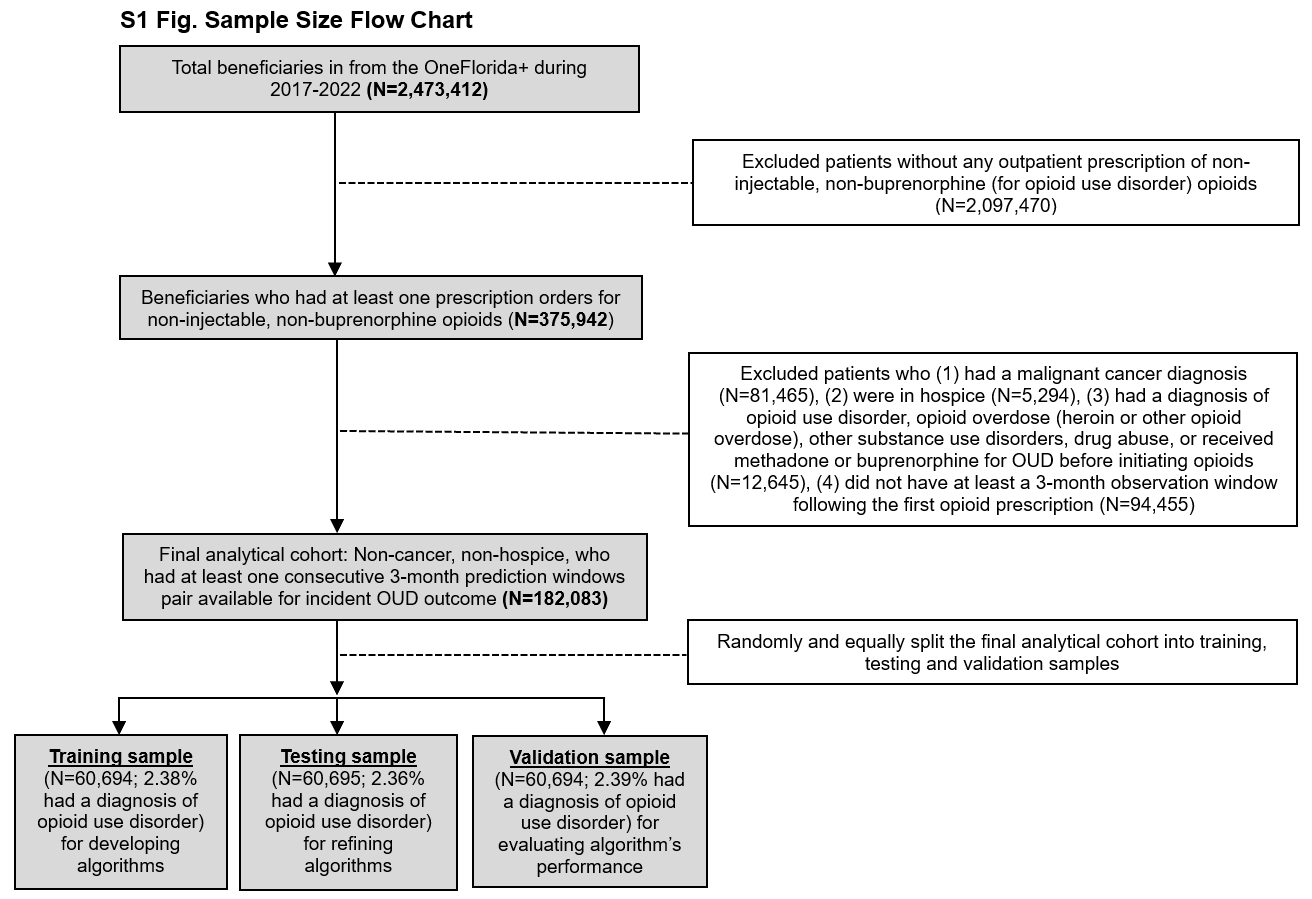


**Figure S2.**


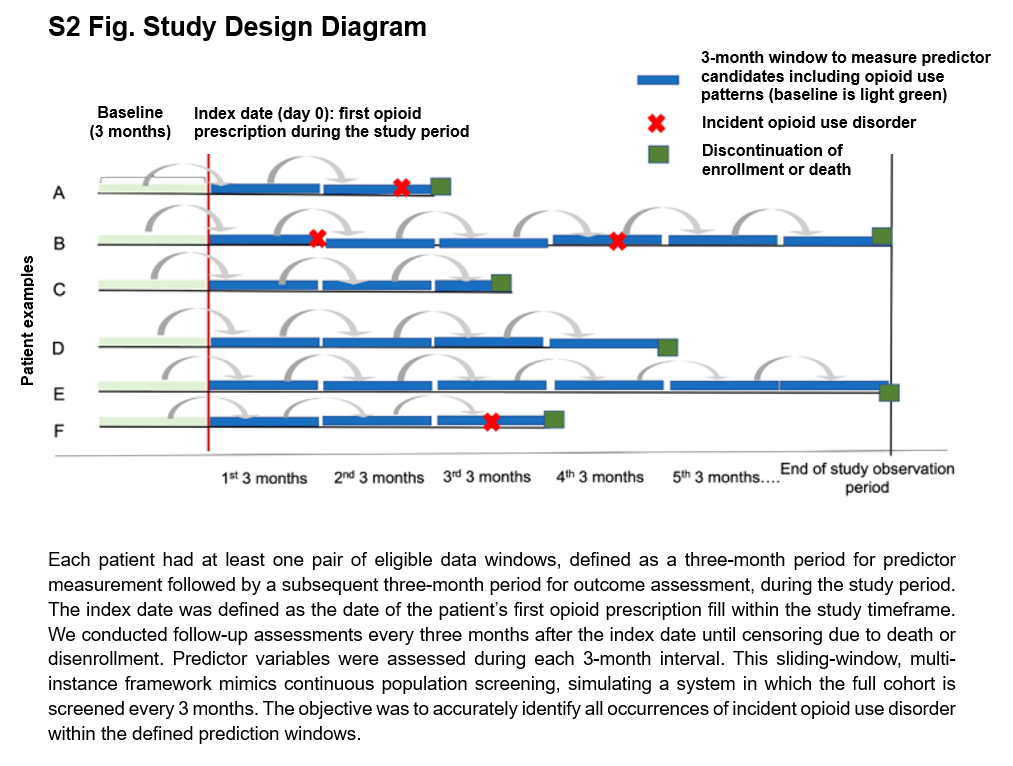


**Figure S3.**





**Figure S4.**


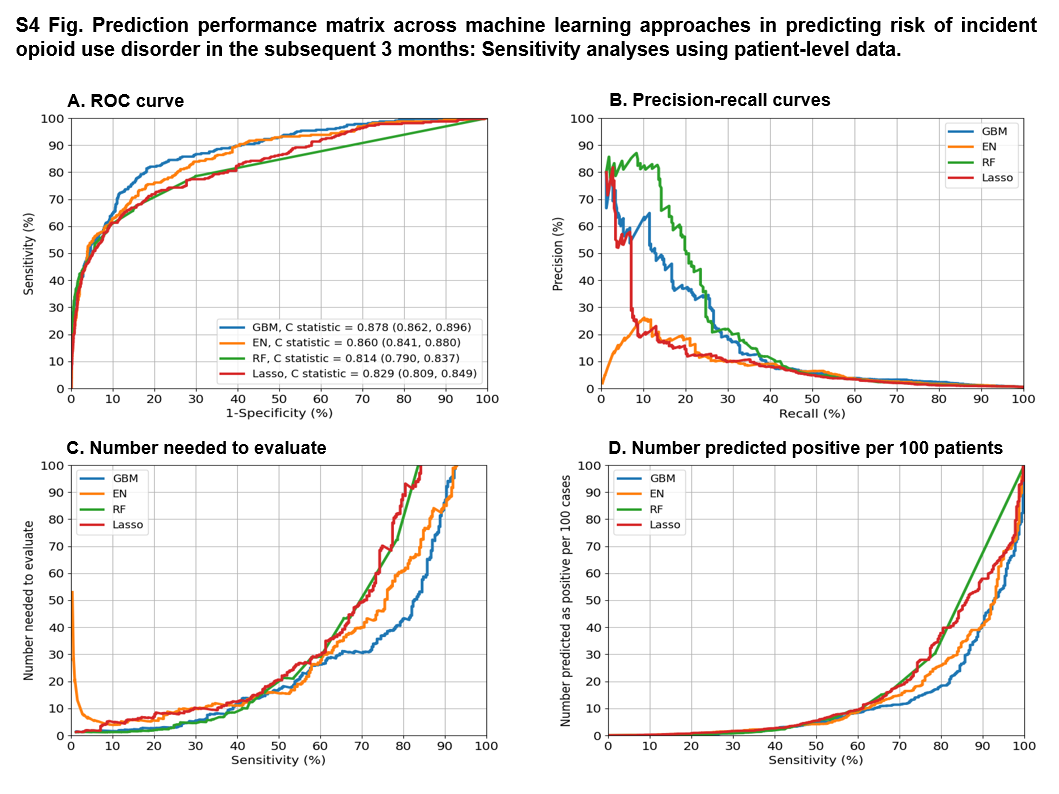


**Figure S5.**


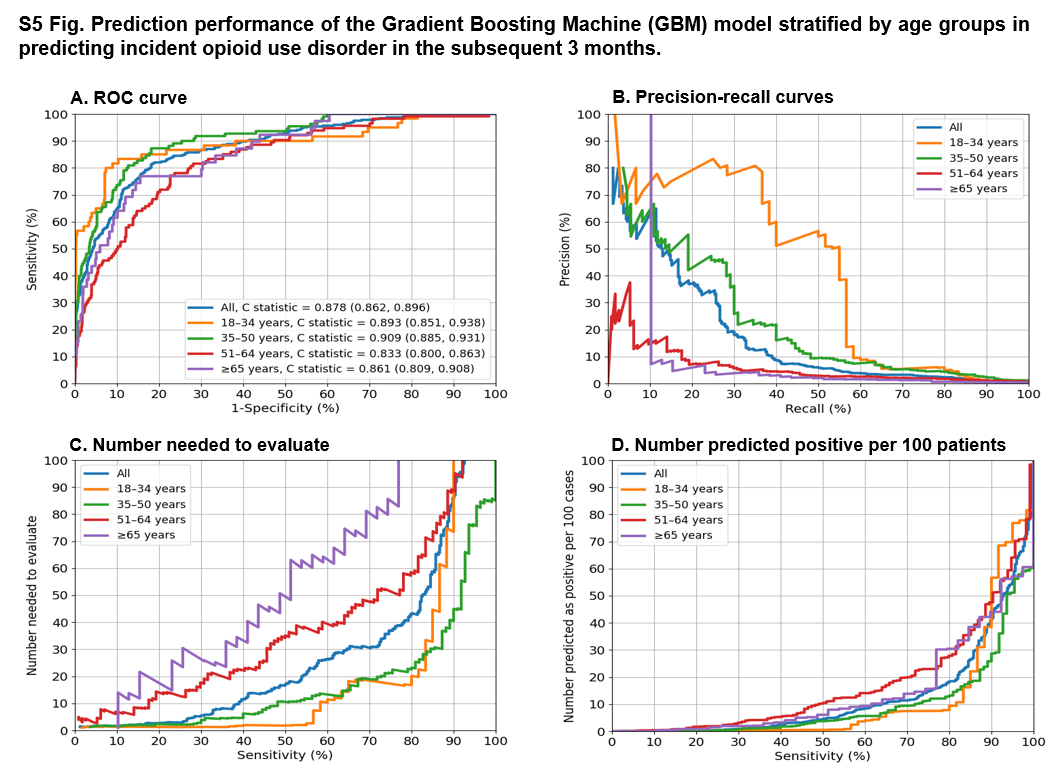


**Figure S6.**


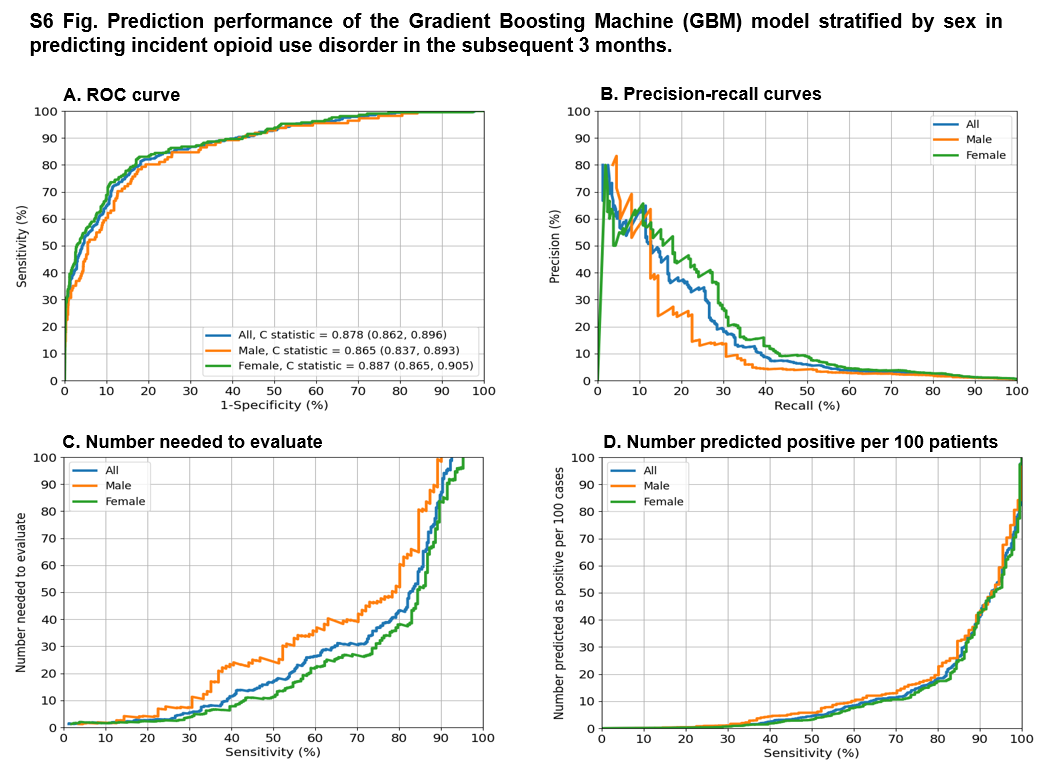


**Figure S7.**


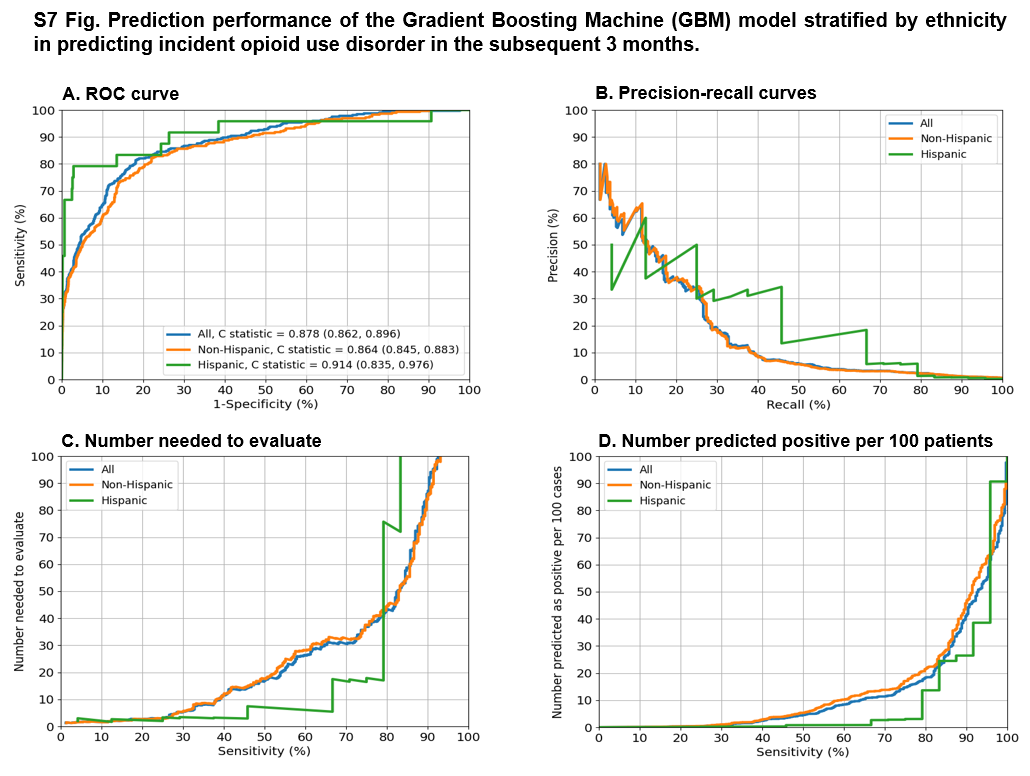


**Figure S8.**


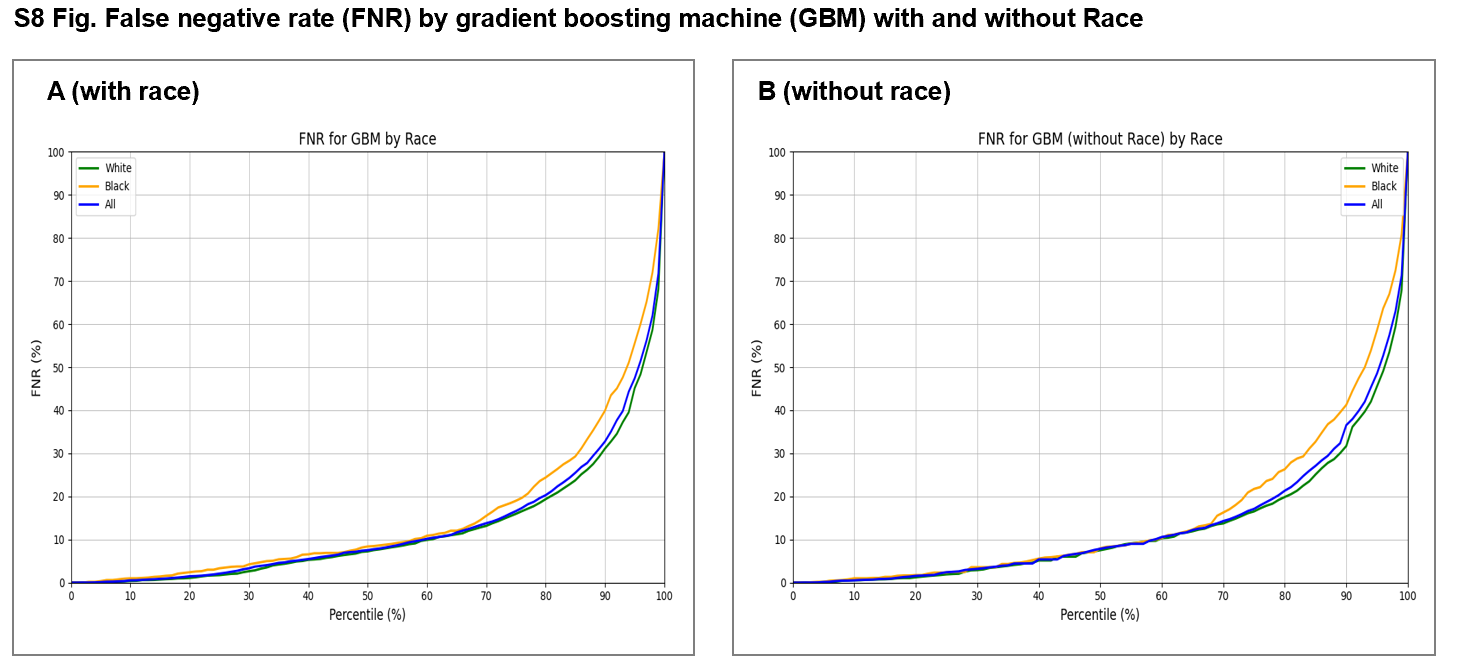


**Figure S9.**


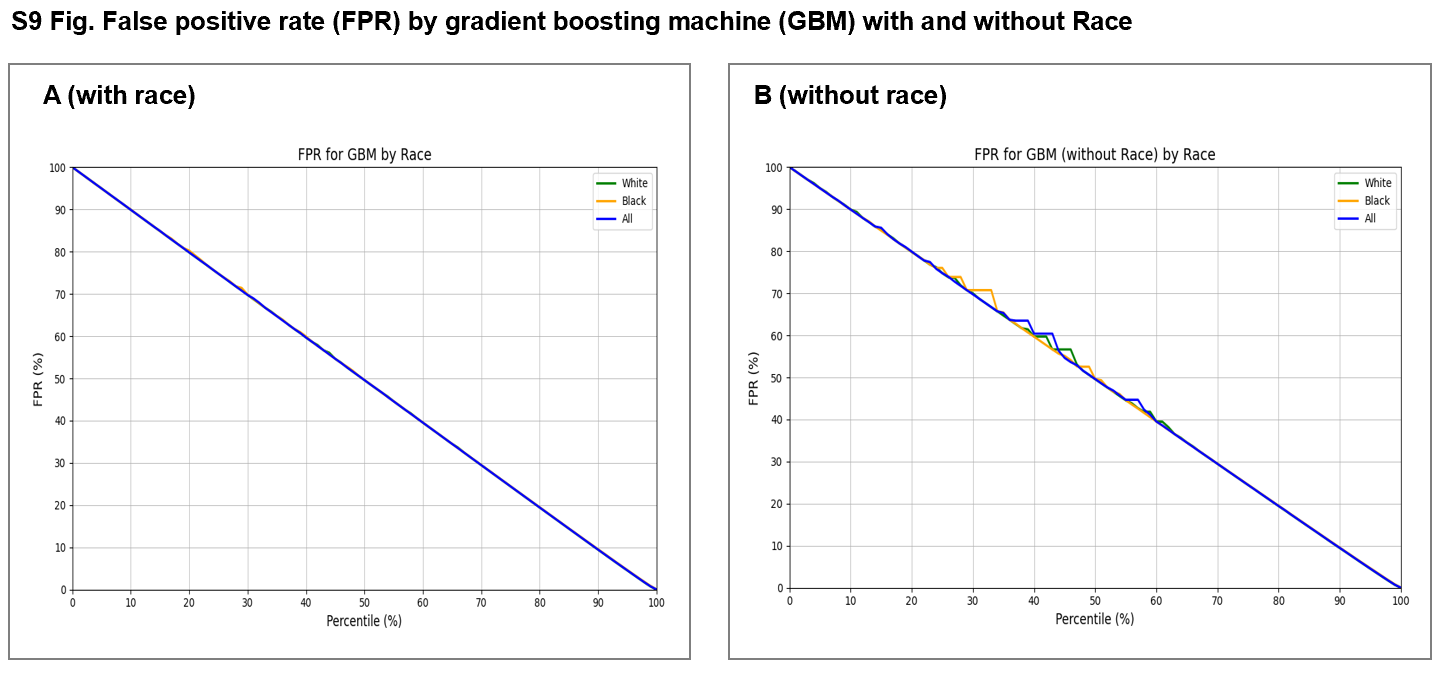


**Figure S10.**


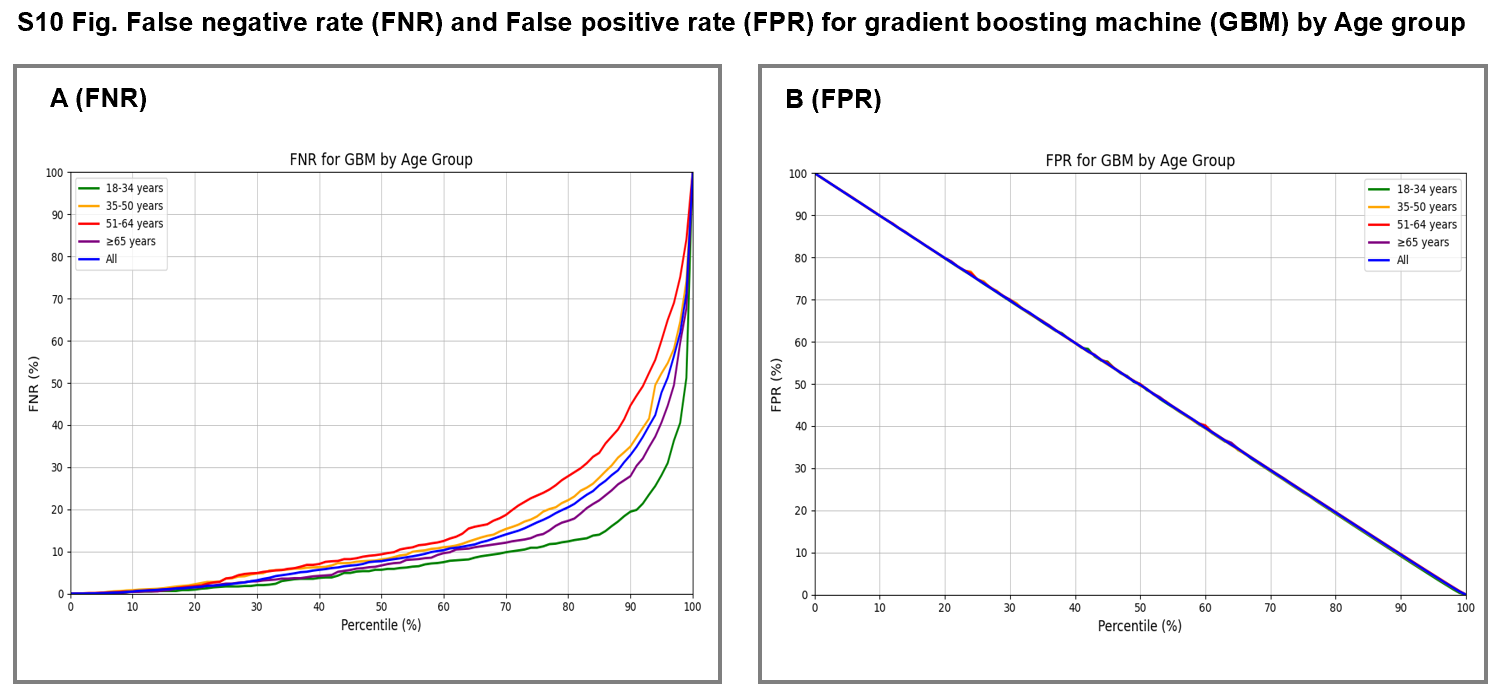


**Figure S11.**


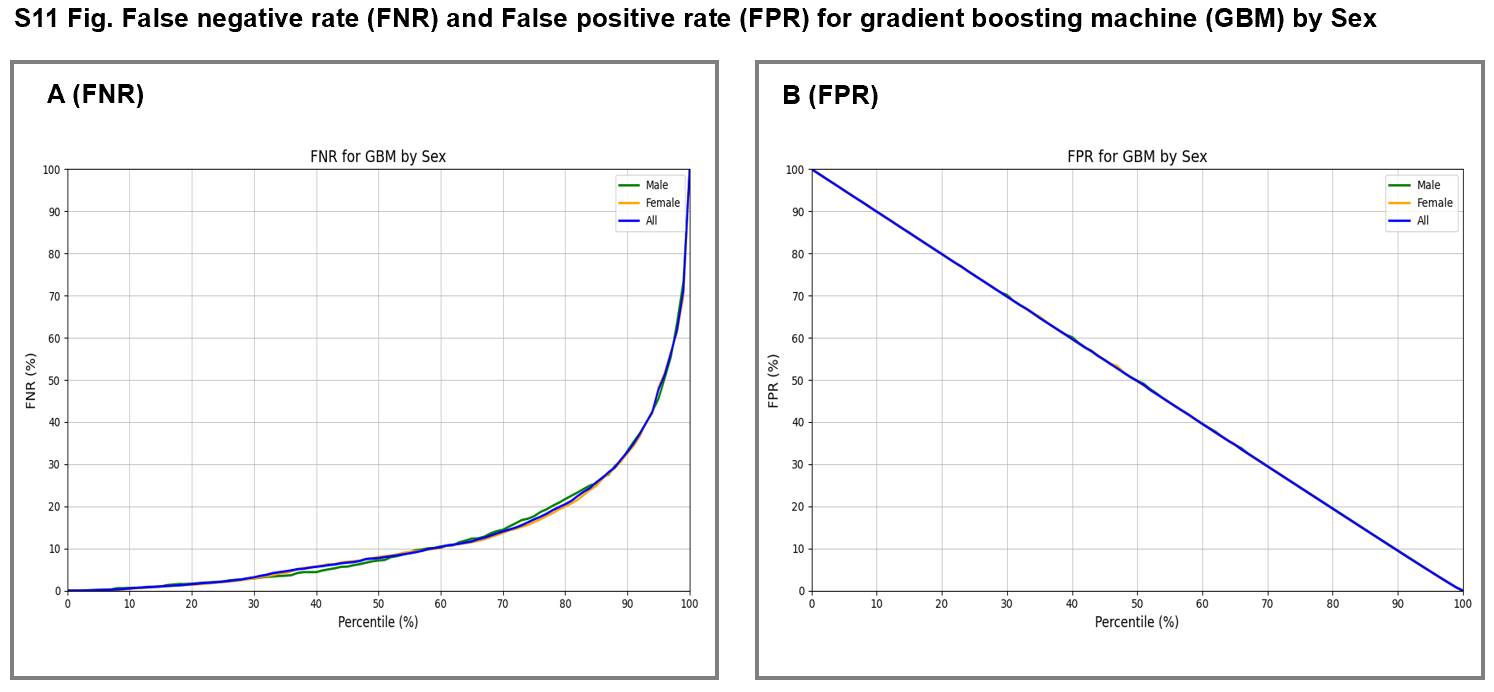


**Figure S12.**


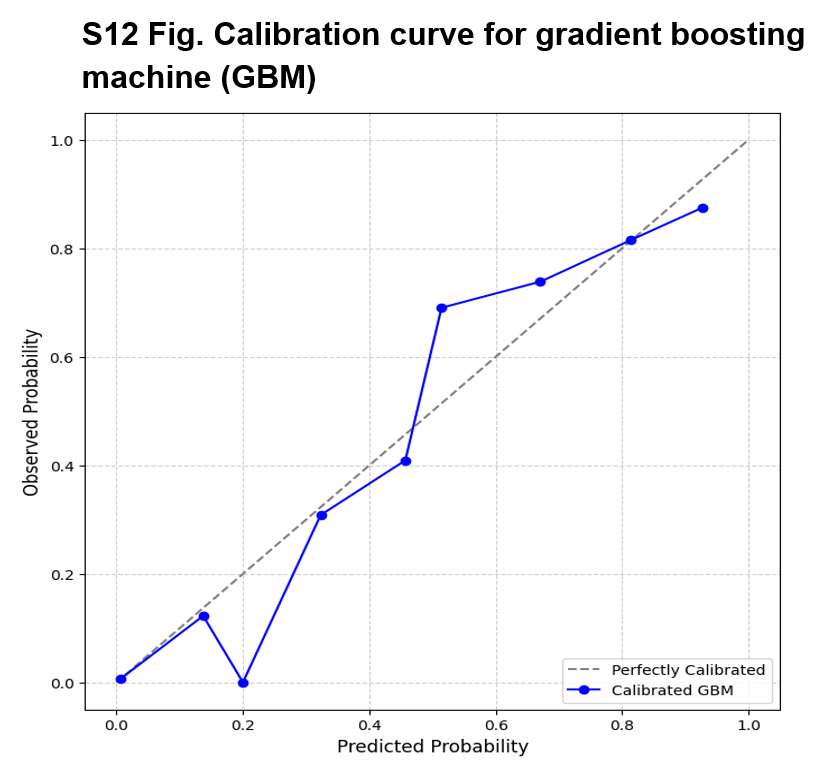


**Figure S13.**


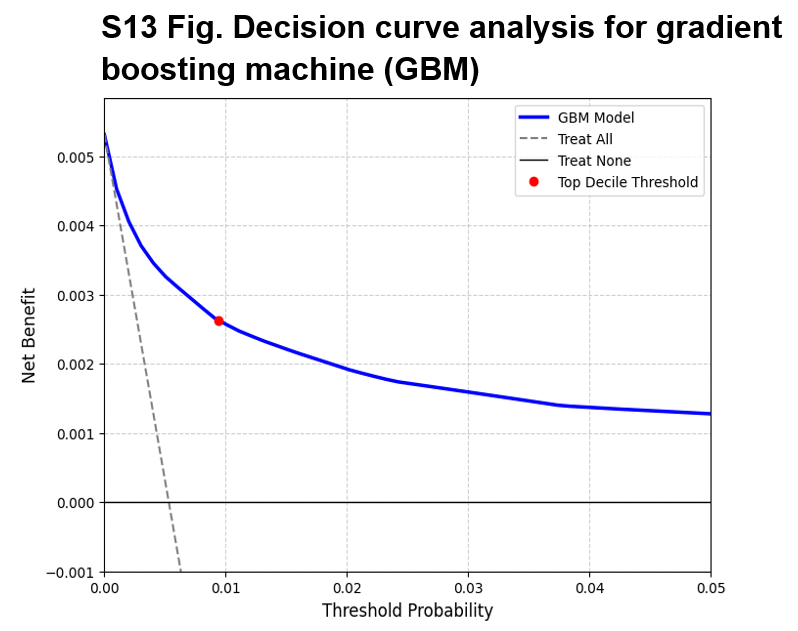


**Figure S14.**


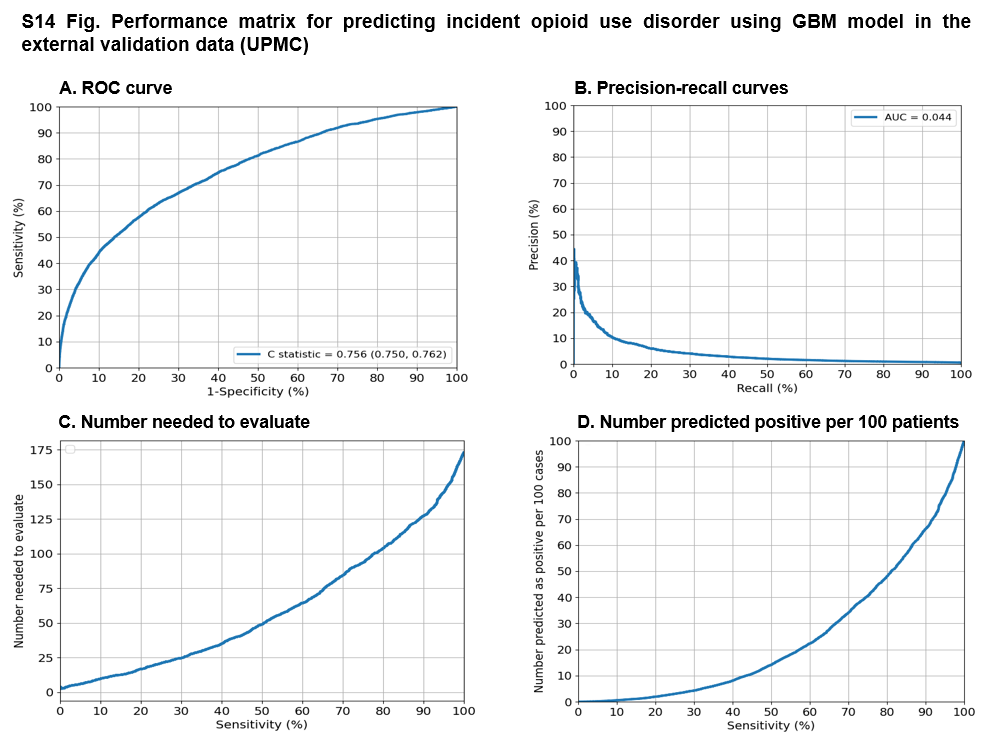


**Figure S15.**


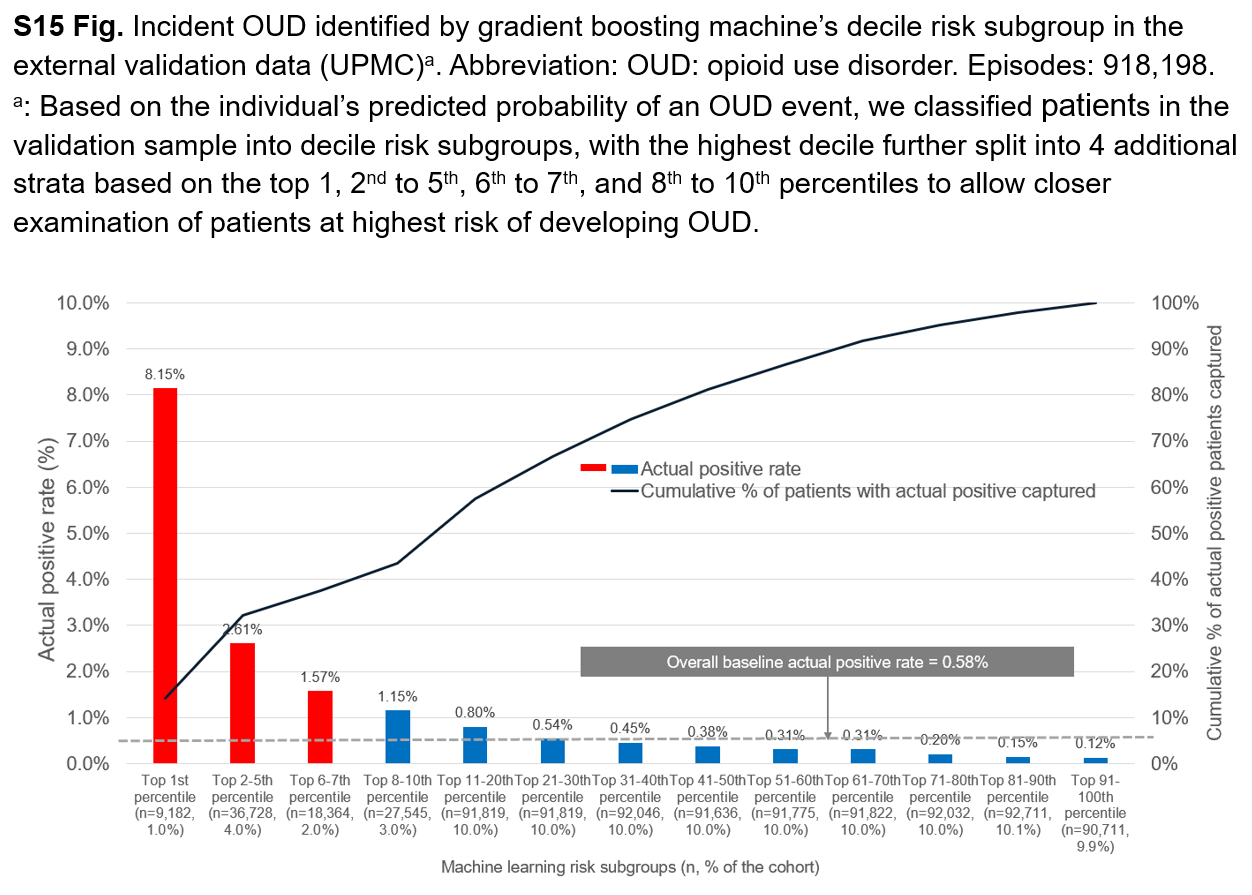


**Figure S16.**


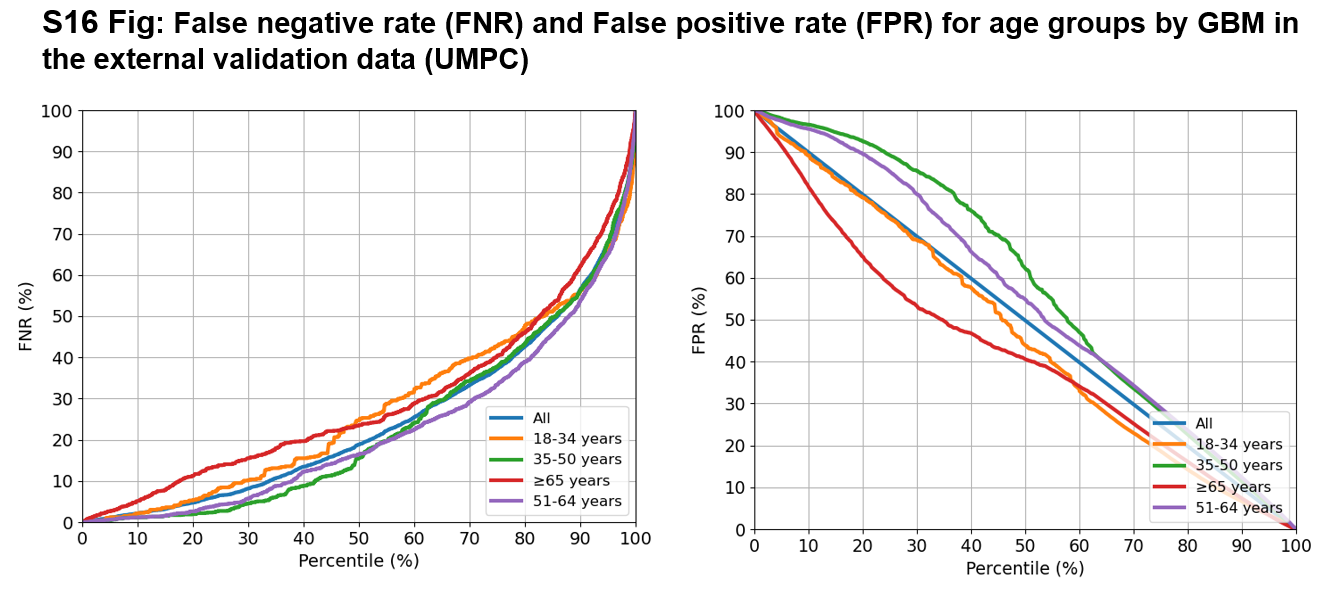


**Figure S17.**


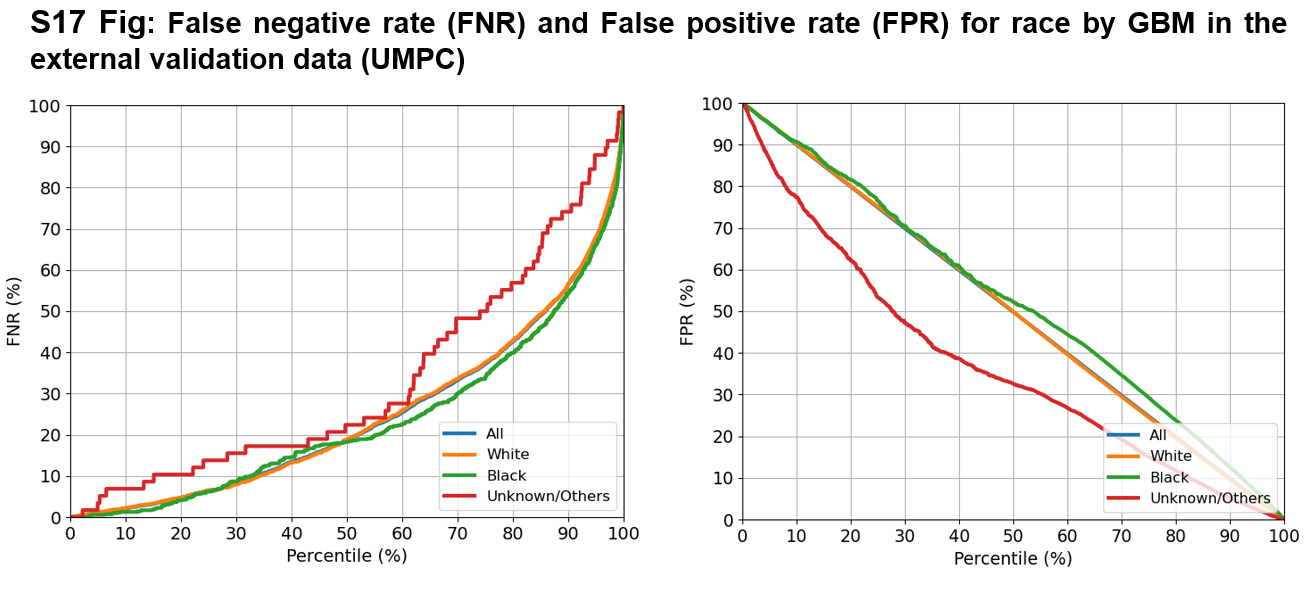


**Figure S18.**


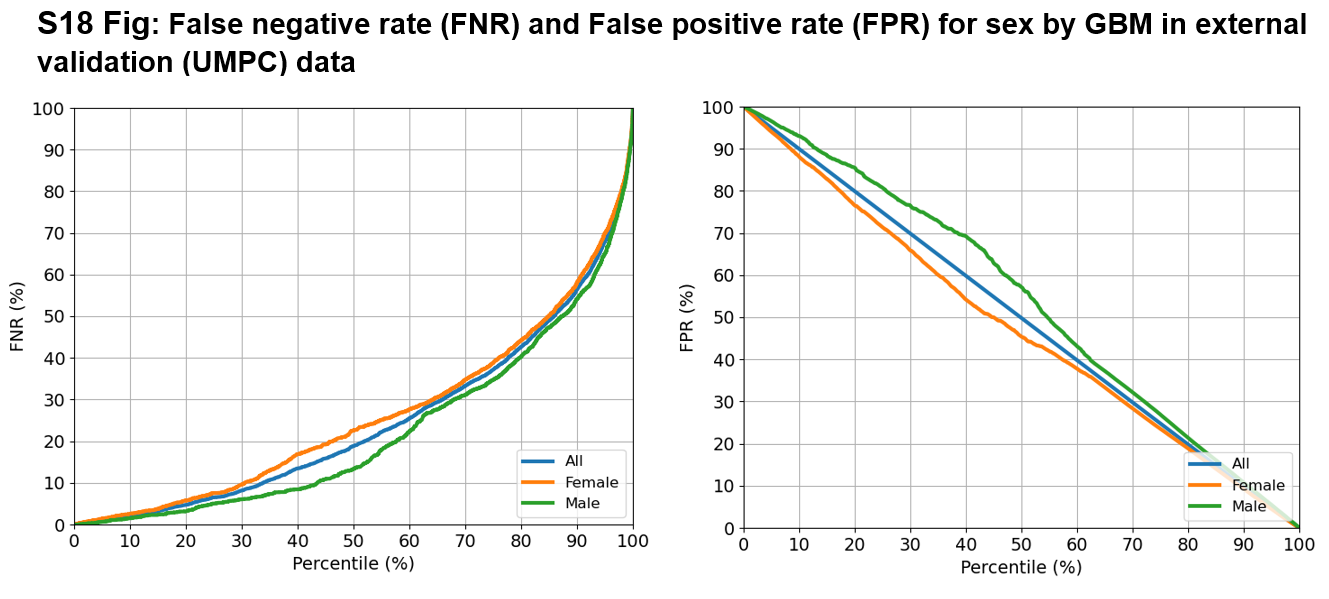

Supplement: Multimedia Appendix 1 [file jmir-v28-e79482-s001.docx]
