# Supplementary material for: Developing and Validating a Machine Learning Algorithm to Predict the Risk of Incident Opioid Use Disorder Among OneFlorida+ Patients: Prognostic Modeling Study
Source: J Med Internet Res. 2026 Mar 5;28:e79482. doi: 10.2196/79482 (PMC12978897; doi:10.2196/79482)
Supplement: Multimedia Appendix 2 [file jmir-v28-e79482-s002.docx]

**Table S1.** Diagnosis codes for the exclusion of patients with malignant cancers based on the National Committee for Quality Assurance (NCQA)’s Opioid Measures in 2018 Healthcare Effectiveness Data and Information Set (HEDIS)

| **ICD-9 codes** | **ICD-10 codes** |
| --- | --- |
| 140.x (x=0, 1, 3, 4, 5, 6, 8, 9), 141.x (x=0, to 6, 8 and 9), 142.x (x=0, 1, 2, 8, 9), 143.x (x=0, 1, 8, 9), 144.x (x=0, 1, 8, 9), 145.x (x=0 to 6, 8, and 9), 146.x (x=0 to 9), 147.x (x=0, 1, 2, 3, 8, 9), 148.x (x=0, 1,2, 3, 8, 9), 149.x (x=0, 1, 8, 9), 150.x (x=0, 1, 2, 3, 4, 5, 8, 9), 151.x (x=0, 1, 2, 3, 4, 5, 6. 8, 9), 152.x (x=0, 1, 2, 3, 8, 9), 153.x (x=0 to 9), 154.x (x=0, 1, 2, 3, 8), 155.x (x=0, 1, 2), 156.x (x=0, 1, 2, 8, 9), 156.x (x=1, 2, 8, 9), 157.x (x=0, 1, 2, 3, 4, 8, 9), 158.x (x=0, 8, 9), 159.x (x=0, 1, 8, 9), 160.x (x=0 to 5, 8, 9), 161.x (x=0, 1, 2, 3, 8, 9), 162.x (x=0, 2, 3, 4, 5, 8, 9), 163.x (x=0, 1, 8, 9), 164.x (x=0, 1, 2, 3, 8, 9), 165.x (x=0, 8, 9), 170.x (x=0 to 9), 171.x (x=0, 2, 3, 4, 5, 6, 7, 8, 9), 172.x (x=0 to 9), 174.x (x=0 to 6, 8, 9), 175.0, 175.9, 176.x (x=0 to 5, 8, 9), 179, 180.x (x=0, 1, 8, 9), 181, 182.x (x=0, 1, 8), 183.x (x=0, 2, 3, 4, 5, 8, 9), 184.x (x=0, 1, 2, 3, 4, 8, 9), 185, 186.0, 186.9, 187.x (x=1 to 9), 188.x (x=0 to 9), 189.x (x=0, 1, 2, 3, 4, 8, 9), 190.x (x=0 to 9), 191.x (x=0 to 9), 192.x (x=0, 1, 2, 3, 8, 9), 193, 194.x (x=0, 1, 3, 4, 5, 6. 8, 9), 195.x (x=0 to 5, 8), 196.x (x=0 to 3, 5, 6, 8, 9), 197.x (x=0 to 8), 198.x (x=0 to 7), 198.81, 198.82, 198.89, 199.x (x=0, 1, 2), 200.0x (x=0 to 8), 200.1x (x=0 to 8), 200.2x (x=0 to 8), 200.3x (x=0 to 8), 200.4x (x=0 to 8), 200.5x (x=0 to 8), 200.6x (x=0 to 8), 200.7x (x=0 to 8), 200.8x (x=0 to 8), 201.0x (x=0 to 8), 201.1x (x=0 to 8), 201.2x (x=0 to 8), 201.4x (x=0 to 8), 201.5x (x=0 to 8), 201.6x (x=0 to 8), 201.7x (x=0 to 8), 201.9x (x=0 to 8), 202.0x (x=0 to 8), 202.1x (x=0 to 8), 202.2x (x=0 to 8), 202.3x (x=0 to 8), 202.4x (x=0 to 8), 202.5x (x=0 to 8), 202.6x (x=0 to 8), 202.7x (x=0 to 8), 202.8x (x=0 to 8), 202.9x (x=0 to 8), 203.0x (x=0 to 2), 203.1x (x=0 to 2), 203.8x (x=0 to 2), 204.0x (x=0 to 2), 204.1x (x=0 to 2), 204.2x (x=0 to 2), 204.8x (x=0 to 2), 204.9x (x=0 to 2), 205.0x (x=0 to 2), 205.1x (x=0 to 2), 205.2x (x=0 to 2), 205.3x (x=0 to 2), 205.8x (x=0 to 2), 205.9x (x=0 to 2), 206.0x (x=0 to 2), 206.1x (x=0 to 2), 206.2x (x=0 to 2), 206.8x (x=0 to 2), 206.9x (x=0 to 2), 207.0x (x=0 to 2), 207.1x (x=0 to 2), 207.2x (x=0 to 2), 207.8x (x=0 to 2), 208.0x (x=0 to 2), 208.1x (x=0 to 2), 208.2x (x=0 to 2), 208.8x (x=0 to 2), 208.9x (x=0 to 2), 209.0x (x=0 to 3), 209.1x (x=0 to 7), 209.2x (x=0 to 7, 9), 209.3x (x=0 to 6), 209.7x (x=0 to 5, 9). | C00.x, C01, C02.x (x=0, 1, 2, 3, 4, 8, 9), C03.x (x=0, 1, 9), C04.0,  C04.1, C04.8, C04.9, C05.0, C05.1, C05.2, C05.8, C05.9, C06.0, C06.1,  C06.2, C06.80, C06.89, C06.9, C07, C08.0, C08.1, C08.9, C09.0, C09.1,  C09.8, C09.9, C10.x (x=0, 1, 2, 3, 4, 8, 9), C11.x (x=0, 1, 2, 3, 8, 9), C12, C13.x (x=0, 1, 2, 3, 8, 9), C14.0, C14.2, C14.8, C15.3, C15.4, C15.5, C15.8, C15.9, C16.x (x=0, 1, 2, 3, 4, 5, 6, 8, 9), C17.x (x=0, 1, 2, 3, 8, 9),  C18.x, C19, C20, C21.x (x=0, 1, 2, 8), C22.x (x=0, 1, 2, 3, 4, 7, 8, 9), C23, C24.x (x=0, 1, 8, 9), C24.1, C24.8, C24.9, C25.x (x=0, 1, 2, 3, 4, 7, 8, 9), C26.0, C26.1, C26.9, C30.0, C30.1, C31.x (x=0, 1, 2, 3, 8, 9), C32.x (x=0, 1, 2, 3, 8, 9), C33, C34.00, C34.01, C34.02, C34.10, C34.11, C34.12, C34.2, C34.30, C34.31, C34.32, C34.80, C34.81, C34.82, C34.90, C34.91, C34.92, C37, C38.x (x=0, 1, 2, 3, 4, 8), C39.0, C39.9, C40.x0 (x=0, 1, 2, 3, 8, 9), C40.x1 (x=0, 1, 2, 3, 8, 9), C40.02 (x=0, 1, 2, 3, 8, 9), C41.x (x=0, 1, 2, 3, 4, 9), C43.0, C43.10, C43.11, C43.12, C43.20, C43.21, C43.22, C43.30, C43.31, C43.39, C43.4, C43.51, C43.52, C43.59, C43.60, C43.61, C43.62, C43.70, C43.71, C43.72, C43.8, C43.9, C45.0, C45.1, C45.2, C45.7, C45.9, C46.0, C46.1, C46.2, C46.3, C46.4, C46.50, C46.51, C46.52, C46.7, C46.9, C47.0, C47.10, C47.11, C47.12, C47.20, C47.21, C47.22, C47.3, C47.4, C47.5, C47.6, C47.8, C47.9, C48.0, C48.1, C48.2, C48.8, C49.0, C49.10, C49.11, C49.12, C49.20, C49.21, C49.22, C49.x (x=3, 4, 5, 6, 8, 9), C49.Ax (x=0, 1, 2,3 ,4, 5, 9), C4A.0, C4A.1x (x=0, 1, 2), C4A.2x (x=0, 1, 2),  C4A.3x (x=0, 1, 9), C4A.4, C4A.5x (x=1, 2, 9), C4A.6x (x=0, 1, 2), C4A.7x (x=0, 1, 2), C4A.8, C4A.9, C50.x11 (x=0, 1, 2, 3, 4, 5, 6, 8, 9), C50.x12 (x=0, 1, 2, 3, 4, 5, 6, 8, 9), C50.x19 (x=0, 1, 2, 3, 4, 5, 6, 8, 9), C50.x21 (x=0, 1, 2, 3, 4, 5, 6, 8, 9), C50.x22 (x=0, 1, 2, 3, 4, 5, 6, 8, 9), C50.x29 (x=0, 1, 2, 3, 4, 5, 6, 8, 9), C51.x (x=0, 1, 2, 8, 9), C52, C53.x (x=0, 1, 3, 8, 9), C54.x (x=2, 3, 8, 9), C55, C56.1, C56.2, C56.9, C57.x0 (x=0, 1, 2), C57.x1 (x=0, 1, 2), C57.02 (x=0, 1, 2), C57.3, C57.4, C57.7, C57.8, C57.9, C58, C60.x (x=0, 1, 2, 8, 9), C61, C62.0x (x=0, 1, 2), C62.1x (x=0, 1, 2), C62.9x (x=0, 1, 2), C63.0x (x=0, 1, 2), C63.1x (x=0, 1, 2), C63.2, C63.7, C63.8, C63.9, C64.1, C64.2, C64.9, C65.1, C65.2, C65.9, C66.1, C66.2, C66.9, C67.x (x=0 to 9), C68.x (x=0, 1, 8, 9), C69.x0 (x=0, 1, 2, 3, 4, 5, 6, 8, 9), C69.x1 (x=0, 1, 2, 3, 4, 5, 6, 8, 9), C69.x2 (x=0, 1, 2, 3, 4, 5, 6, 8, 9), C70.0, C70.1, C70.9, C71.x, C72.0, C72.1, C72.x0 (x=2 to 5), C72.x1 (x=2 to 4), C72.x2 (x=2 to 4), C72.59, C72.9, C73, C74.0x (x=0, 1, 2), C74.1x (x=0, 1, 2), C74.9x (x=0, 1, 2), C75.x (x=0, 1, 2, 3, 4, 5, 8, 9). C76.x (x=0 to 3), C76.4x (x=0, 1, 2), C76.5x (x=0, 1, 2, 8), C77.x (x=0, 1, 2, 3, 4, 5, 8, 9), C78.0x (x=0, 1, 2), C78.1, C78.2, C78.30, C78.39, C78.4, C78.5, C78.6, C78.7, C78.80, C78.89, C79.0x (x=0, 1, 2), C79.10, C79.11, C79.19, C79.2, C79.31, C79.32, C79.40, C79.49, C79.51, C79.52, C79.6x (x=0, 1, 2), C79.7x (0, 1, 2), C79.8x (x=1, 2, 9), C79.9, C7A.00, C7A.010, C7A.011, C7A.012, C7A.019, C7A.02x (x=0 to 6, and 9), C7A.09x (x=0 to 6 and 8), C7A.1, C7A.8, C7B.0x (x=0, 1, 2, 3, 4, 9), C7B.1, C7B.8, C81.0x, C81.1x, C81.2x, C81.3x, C81.4x, C81.7x, C81.9x, C82.0x, C82.1x, C82.2x, C82.3x, C82.4x, C82.5x, C82.6x, C82.8x, C82.9x, C83.0x, C83.1x, C83.3x, C83.5x, C83.7x, C83.8x, C83.9x, C84.0x, C84.1x, C84.4x, C84.6x, C84.7x, C84.9x, C84.Ax, C84.Zx, C85.1x, C85.2x, C85.8x, C85.9x, C86.x (x=0 to 6), C88.x (x=0, 2, 3, 4, 8, 9), C90.x0 (x=0 to 3), C90.x1 (x=0 to 3), C90.x2 (x=0 to 3), C91.x0 (x=A, Z, 0, 1, 3, 4, 5, 6, 9), C91.x1 (x=A, Z, 0, 1, 3, 4, 5, 6, 9), C92.x2 (x=A, Z, 0, 1, 3, 4, 5, 6, 9), C92.9x (x=0, 1, 2), C92.Ax (x=0, 1, 2), C92.Zx (x=0, 1, 2), C93.0x (x=0, 1, 2), C93.1x (x=0, 1, 2), C93.3x (x=0, 1, 2), C93.9x (x=0, 1, 2), C93.Zx (x=0, 1, 2), C94.0x (x=0, 1, 2), C94.2x (x=0, 1, 2), C94.3x (x=0, 1, 2), C94.4x (x=0, 1, 2), C94.6, C94.8x (x=0, 1, 2), C95.0x (x=0, 1, 2), C95.1x (x=0, 1, 2), C95.9x (x=0, 1, 2), C96.x (x=0, 2, 4, 5, 6, 9, A, Z) |

**Table S2.** Diagnosis codes for identifying incident opioid use disorder

| **Conditions** | **ICD-9 codes** | **ICD-10 codes** |
| --- | --- | --- |
| Incident opioid use disorder | 304.0X, 304.7X, 305.5X | F11.1X and F11.2X, excluding F11.11 (opioid-related disorders in remission) and F11.21 (opioid dependence in remission) |

**Table S3.** Summary of predictor candidates (n=183) measured in 3-month windows for predicting incident opioid use disorder ^a^

| **Patterns of prescription opioid use^b^** | **Patterns of non-opioid prescription use** | **Beneficiaries sociodemographics** | **Health status factors** | **Opioid prescriber-level variables^d^** |
| --- | --- | --- | --- | --- |
| - Average opioid daily dose in MME^c^ - Cumulative MME - Cumulative duration for any opioids, SAO, and LAO - Duration of longest continuous use for any opioids, SAO, and LAO - No. fills of any opioids, SAO, and LAO - No. standardized 30-day prescriptions for any opioids, SAO, and LAO - Cumulative duration of 30-day use of any opioids, SAO, and LAO - No. fills by opioid ingredient and type (e.g., any fentanyl, SAO-type fentanyl, LAO-type fentanyl) - Type of opioids by Schedule and SAO/LAO (e.g., SAO, Schedule I only) - No. unique opioid prescribers - No. unique pharmacies - No. early refills for opioids - Cumulative overlapping days of early refills - Use of injectible opioids or antitussive opioids | - No. BZD fills - No. muscle relaxants fills - Cumulative overlapping days of concurrent opioid and BZD use - Cumulative overlapping days of concurrent opioid and muscle relaxants use - Cumulative overlapping days of concurrent opioid, BZD and muscle relaxants use - Cumulative duration of buprenorphine for opioid use disorder - Cumulative duration of naltrexone - No. gabapentinoid fills - Cumulative duration of gabapentinoid use - No. antidepressants fills - Cumulative duration of antidepressant use - No. average monthly non-opioid prescriptions - No. naltrexone fills | - Age - Sex - Race - Ethnicity - State of residence - County of residence - Zip code of residence - Type of county of residence (metro vs. non-metro) | - No. outpatient visits - No. ED visits - No. inpatient visits - History of prescription opioid overdose - History of heroin overdose - Non-opioid drug use disorders - Other non-opioid SUD or alcohol use disorders - Alcohol use disorders - History of urine drug tests - History of SUD counseling - Adjustment disorders - Personality disorders - Psychoses - Delusional disorders - Schizophrenia - Mood disorders - Anxiety disorders - Alcohol-induced mental disorders - Drug-induced mental or sleep disorders - Other mental health disorders - Osteoarthritis - Rheumatoid arthritis - Back pain - Neck pain - Headache or migraine - Temporomandibular disorder pain - Abdominal pain or hernia - Chest pain - Kidney or gall bladder stones - Menstrual or genital reproductive pain - Fractures, concussion, injuries - Fibromyalgia - Internal orthopedic device implant/graft - Other pain conditions - Surgical procedures (e.g., ischemic heart diseases) - Diseases of musculoskeletal system and connective tissues - Neuropathies (excluding alcoholic, drug, and optic-related) - Ischemic heart disease - HIV/AIDS - Elixhauser index and individual categories | - Prescriber’s sex - Prescriber’s categories - Average monthly opioid prescribing volume - Average monthly opioid prescribing dose in MME - Average monthly patients receiving opioids |

Abbreviations: AHRF: Area Health Resources Files; BZD: benzodiazepines; LAO: long-acting opioids; MME: morphine milligram equivalent; No: Number of; SAO: short-acting opioids; SUD: substance use disorders;
^a:^ Details for the operational definitions for each variable and corresponding diagnosis and procedure codes and National Drug Codes can be provided per request to the corresponding author.
^b:^ We used an “as-prescribed” approach that assumes patients taking all prescribed opioids on the schedule recommended by their clinicians. (Bohnert AS et al. JAMA. 2011;305(13):1315-21. doi: 10.1001/jama.2011.370.) Patients who received refills for the same drug at the same dose and schedule while still having opioid prescriptions within three days from a prior fill were assumed to have taken the medication from the prior fill before taking medication from the second fill. (Gellad WF et al. Am J Public Health. 2018;108(2):248-255. doi: 10.2105/AJPH.2017.304174.)

^c:^ We calculated morphine milligram equivalent (MME) for each opioid prescription, defined by the quantity dispensed multiplied by the strength in milligrams, multiplied by a conversion factor. **(**Bohnert AS et al. JAMA. 2011;305(13):1315-21. doi: 10.1001/jama.2011.370.) For each person, the average daily MME during the 3-month window was calculated by summing MMEs across all opioids and dividing by the number of days supplied.

^d:^ Prescribers were identified by their National Provider Identifiers. Primary opioid prescribers were defined as the prescribers who dominantly prescribed the most opioid prescriptions. If patients only had two opioid prescriptions, then the first prescriber was considered as the primary prescriber.

^e^: AHRF variables (<https://data.hrsa.gov/topics/health-workforce/ahrf>), area deprivation index (<https://www.hipxchange.org/ADI>), and county-health ranking variables (<http://www.countyhealthrankings.org/explore-health-rankings/use-data>) are publicly available and downloadable.
f: Methadone for opioid use disorder was identified using the procedure codes (H0020, J1230) and buprenorphine for opioid use disorder was identified from prescription sublingual buprenorphine or buprenorphine/naloxone using NDC codes.

**Table S4.** Prediction performance measures for predicting incident opioid use disorder, across machine learning methods

with varying sensitivity and specificity.

| **Methods** | **Score threshold**  **(range 0-100)^a^** | **Predicted OUD (%)** | **Sensitivity (%)** | **Specificity (%)** | **PPV (%)** | **NPV (%)** | **NNE** |  |
| --- | --- | --- | --- | --- | --- | --- | --- | --- |
| **GBM** |  |  |  |  |  |  |  |  |
| **Sensitivity** | | | | | | | |  |
| 100% | 3.02 | 99.64 | 100 | 0.37 | 0.90 | 100 | 111 |  |
| 99% | 11.66 | 84.57 | 99.01 | 15.56 | 1.05 | 99.94 | 95 |  |
| 98% | 14.67 | 74.82 | 97.99 | 25.39 | 1.18 | 99.93 | 85 |  |
| 97% | 16.21 | 69.68 | 97.00 | 30.57 | 1.25 | 99.91 | 80 |  |
| 96% | 17.00 | 65.88 | 96.01 | 34.39 | 1.31 | 99.89 | 76 |  |
| 95% | 18.07 | 61.55 | 94.99 | 38.76 | 1.39 | 99.88 | 72 |  |
| 94% | 19.02 | 56.97 | 94.00 | 43.37 | 1.49 | 99.87 | 67 |  |
| 93% | 20.27 | 52.39 | 93.01 | 47.98 | 1.60 | 99.87 | 63 |  |
| 92% | 21.73 | 46.33 | 91.99 | 54.08 | 1.79 | 99.87 | 56 |  |
| 91% | 23.12 | 42.45 | 91.00 | 57.99 | 1.93 | 99.86 | 52 |  |
| 90% | 24.17 | 39.82 | 89.99 | 60.26 | 2.03 | 99.96 | 49 |  |
| **Optimized threshold^b^** | 43.28 | 18.61 | 76.59 | 84.75 | 4.36 | 99.75 | 23 |  |
| **Specificity** | | | | | | | |  |
| 90% | 57.74 | 10.54 | 68.46 | 90 | 6.16 | 99.68 | 16 |  |
| 91% | 59.85 | 9.53 | 67.42 | 91 | 6.37 | 99.68 | 16 |  |
| 92% | 62.16 | 8.51 | 64.78 | 92 | 6.85 | 99.65 | 15 |  |
| 93% | 64.75 | 7.49 | 62.54 | 93 | 7.52 | 99.64 | 13 |  |
| 94% | 67.49 | 6.49 | 60.6 | 94 | 8.41 | 99.62 | 12 |  |
| 95% | 70.56 | 5.48 | 57.57 | 95 | 9.47 | 99.6 | 11 |  |
| 96% | 74 | 4.43 | 51.91 | 96 | 10.55 | 99.55 | 9 |  |
| 97% | 78.2 | 3.39 | 45.87 | 97 | 12.19 | 99.5 | 8 |  |
| 98% | 83.1 | 2.35 | 40.55 | 98 | 15.56 | 99.45 | 6 |  |
| 99% | 88.86 | 1.29 | 33.30 | 99 | 23.25 | 99.39 | 4 |  |
| 100% | 96.8 | 0.00 | 0.00 | 100 | 0.00 | 99.10 | inf |  |
| **EN** |  |  |  |  |  |  |  |  |
| **Sensitivity** | | | | | | | |  |
| 100% | 5.16 | 99.89 | 100 | 0.36 | 0.90 | 100 | 111 |  |
| 99% | 13.84 | 86.82 | 99.01 | 13.29 | 1.03 | 99.93 | 97 |  |
| 98% | 18.59 | 79.27 | 97.99 | 20.90 | 1.11 | 99.91 | 90 |  |
| 97% | 19.35 | 73.66 | 96.92 | 26.55 | 1.18 | 99.89 | 84 |  |
| 96% | 19.85 | 69.61 | 96.01 | 30.63 | 1.24 | 99.88 | 81 |  |
| 95% | 20.45 | 64.79 | 94.99 | 35.48 | 1.32 | 99.87 | 76 |  |
| 94% | 20.94 | 61.12 | 94.00 | 39.18 | 1.38 | 99.86 | 72 |  |
| 93% | 21.57 | 56.75 | 93.01 | 43.58 | 1.48 | 99.85 | 68 |  |
| 92% | 22.51 | 51.39 | 91.99 | 48.98 | 1.61 | 99.85 | 62 |  |
| 91% | 23.47 | 47.28 | 91.00 | 53.12 | 1.73 | 99.85 | 58 |  |
| 90% | 24.55 | 43.56 | 90.01 | 60.26 | 1.86 | 99.84 | 54 |  |
| **Optimized threshold^b^** | 47.16 | 15.42 | 77.65 | 82.74 | 3.93 | 99.76 | 25 |  |
| **Specificity** | | | | | | | |  |
| 90% | 56.87 | 10.53 | 67.55 | 90 | 6.08 | 99.68 | 16 |  |
| 91% | 59.22 | 9.52 | 67.89 | 91 | 6.34 | 99.67 | 16 |  |
| 92% | 61.91 | 8.51 | 64.59 | 92 | 6.83 | 99.65 | 15 |  |
| 93% | 64.9 | 7.50 | 62.5 | 93 | 7.5 | 99.63 | 13 |  |
| 94% | 68.06 | 6.48 | 59.35 | 94 | 8.25 | 99.61 | 12 |  |
| 95% | 71.70 | 5.46 | 56.43 | 95 | 9.30 | 99.59 | 11 |  |
| 96% | 76.01 | 4.43 | 52.27 | 96 | 10.62 | 99.55 | 9 |  |
| 97% | 80.98 | 3.40 | 46.61 | 97 | 12.34 | 99.5 | 8 |  |
| 98% | 86.82 | 2.36 | 40.78 | 98 | 15.59 | 99.45 | 6 |  |
| 99% | 93.34 | 1.28 | 32.41 | 99 | 22.76 | 99.38 | 4 |  |
| 100% | 95.20 | 0.00 | 0.00 | 100 | 0.00 | 99.10 | inf |  |
| Abbreviations: GBM: gradient boosting machine; INF: infinity; N/A: not able to calculate; NNE: number needed to evaluate; NPV: negative predictive values; PPV: positive predictive values; EN: Elastic net ^a^: Scores were calculated by predicted probability multiplied by 100. Score threshold refers to the score used to classify or predict individuals with OUD (i.e., ≥ the threshold) vs. non-OUD (i.e., <threshold) ^b^: Optimized threshold was calculated by the Youden Index to achieve balanced sensitivity and specificity. | | | | | | | | |

**Table S5.** Racial distribution of true positive cases across gradient boosting machine (GBM)–predicted risk deciles for incident opioid use disorder.

| **Risk group** | **Black** | **Others** | **White** | **All** | **TP-Black** | **TP-Others** | **TP-White** | **TP** |
| --- | --- | --- | --- | --- | --- | --- | --- | --- |
| 91^th^-100^th^ | 1712 | 1619 | 8630 | 11961 | 0 | 0 | 3 | 3 |
| 81^th^-90^th^ | 1158 | 672 | 3694 | 5524 | 2 | 0 | 2 | 4 |
| 71^th^-80^th^ | 1068 | 625 | 3409 | 5102 | 2 | 0 | 4 | 6 |
| 61^th^-70^th^ | 780 | 414 | 2175 | 3369 | 0 | 1 | 1 | 2 |
| 51^th^-60^th^ | 856 | 329 | 2272 | 3457 | 4 | 0 | 2 | 6 |
| 41^th^-50^th^ | 1054 | 370 | 2940 | 4364 | 2 | 1 | 8 | 11 |
| 31^th^-40^th^ | 1524 | 377 | 4400 | 6301 | 3 | 0 | 7 | 10 |
| 21^th^-30^th^ | 1830 | 424 | 4696 | 6950 | 4 | 2 | 10 | 16 |
| 11^th^-20^th^ | 1834 | 294 | 4842 | 6970 | 10 | 3 | 34 | 47 |
| 6^th^-10^th^ | 906 | 116 | 2324 | 3346 | 10 | 0 | 54 | 64 |
| 2^th^-5^th^ | 726 | 69 | 2008 | 2803 | 18 | 2 | 82 | 102 |
| Top 1^st^ | 116 | 12 | 419 | 547 | 13 | 4 | 35 | 52 |

Top decile: Total OUD cases: 218 (White: 171 [78.44%], Black: 41 [18.81%], Other: 6 [2.75%])

Top 1^st^ percentile: Total OUD cases: 52 (White: 35 [67.31%], Black: 13 [25%], Other: 4 [7.69%])

Top 5^th^ percentile: Total OUD cases: 154 (White: 117 [75.97%], Black: 31 [20.13%], Other: 6 [3.9%])

**Table S6.** Age group distribution of true positive cases across gradient boosting machine (GBM)-predicted risk deciles for incident opioid use disorder.

| **Risk group** | **18-34**  **years** | **35-50**  **years** | **51-64**  **years** | **≥65**  **years** | **All** | **TP**  **18-34 years** | **TP**  **35-50 years** | **TP**  **51-64 years** | **TP**  **≥65 years** | **TP** |
| --- | --- | --- | --- | --- | --- | --- | --- | --- | --- | --- |
| 91^th^-100^th^ | 2149 | 2445 | 3186 | 4181 | 11961 | 0 | 1 | 1 | 1 | 3 |
| 81^th^-90^th^ | 1161 | 1300 | 1523 | 1540 | 5524 | 0 | 1 | 2 | 1 | 4 |
| 71^th^-80^th^ | 1026 | 1295 | 1515 | 1266 | 5102 | 0 | 0 | 3 | 3 | 6 |
| 61^th^-70^th^ | 741 | 863 | 948 | 817 | 3369 | 1 | 0 | 1 | 0 | 2 |
| 51^th^-60^th^ | 698 | 874 | 1026 | 859 | 3457 | 0 | 5 | 1 | 0 | 6 |
| 41^th^-50^th^ | 843 | 1229 | 1324 | 968 | 4364 | 2 | 3 | 4 | 2 | 11 |
| 31^th^-40^th^ | 1092 | 1813 | 1985 | 1411 | 6301 | 1 | 1 | 4 | 4 | 10 |
| 21^th^-30^th^ | 1073 | 1880 | 2333 | 1664 | 6950 | 2 | 6 | 8 | 0 | 16 |
| 11^th^-20^th^ | 826 | 1931 | 2539 | 1674 | 6970 | 3 | 13 | 21 | 10 | 47 |
| 6^th^-10^th^ | 375 | 924 | 1338 | 709 | 3346 | 20 | 18 | 21 | 5 | 64 |
| 2^th^-5^th^ | 256 | 789 | 1184 | 574 | 2803 | 18 | 35 | 38 | 11 | 102 |
| Top 1^st^ | 69 | 173 | 231 | 74 | 547 | 8 | 29 | 11 | 4 | 52 |

Top 1^st^ percentile: Total OUD cases: 52 (35-50 years: 29 [55.8%], 51-64 years: 11 [21.2%], 18-34 years: 8 [15.4%], 65+ years: 4 [7.7%])

Top 5^th^ percentile: Total OUD cases: 154 (35-50 years: 64 [62.75%], 51-64 years: 49 [48.04%], 18-34 years: 26 [25.49%], 65+ years: 15 [14.71%])

Top decile: Total OUD cases: 218 (35-50 years: 82 [37.61%], 51-64 years: 70 [32.11%], 18-34 years: 46 [21.10%], 65+ years: 20 [9.17%])

**Table S7.** Sex distribution of true positive cases across gradient boosting machine (GBM)-predicted risk deciles for incident opioid use disorder.

| **Risk group** | **Female** | **Male** | **All** | **TP-Female** | **TP-Male** | **TP** |
| --- | --- | --- | --- | --- | --- | --- |
| 91^th^-100^th^ | 7827 | 4134 | 11961 | 2 | 1 | 3 |
| 81^th^-90^th^ | 3685 | 1839 | 5524 | 2 | 2 | 4 |
| 71^th^-80^th^ | 3200 | 1902 | 5102 | 4 | 2 | 6 |
| 61^th^-70^th^ | 2068 | 1301 | 3369 | 2 | 0 | 2 |
| 51^th^-60^th^ | 2228 | 1229 | 3457 | 5 | 1 | 6 |
| 41^th^-50^th^ | 2648 | 1716 | 4364 | 8 | 3 | 11 |
| 31^th^-40^th^ | 3530 | 2771 | 6301 | 5 | 5 | 10 |
| 21^th^-30^th^ | 4093 | 2857 | 6950 | 10 | 6 | 16 |
| 11^th^-20^th^ | 4050 | 2920 | 6970 | 28 | 19 | 47 |
| 6^th^-10^th^ | 1985 | 1361 | 3346 | 47 | 17 | 64 |
| 2^th^-5^th^ | 1563 | 1240 | 2803 | 66 | 36 | 102 |
| Top 1^st^ | 301 | 246 | 547 | 33 | 19 | 52 |

Top 1^st^ percentile: Total OUD cases: 52 (Female: 33 [63.5%], Male: 19 [36.5%])

Top 5^th^ percentile: Total OUD cases: 154 (Female: 99 [64.29%], Male: 55 [35.71%])

Top decile: Total OUD cases: 218 (Female: 146 [66.97%], Male: 72 [33.03%])
