# Supplementary material for: Developing and Validating a Machine Learning Algorithm to Predict the Risk of Incident Opioid Use Disorder Among OneFlorida+ Patients: Prognostic Modeling Study
Source: J Med Internet Res. 2026 Mar 5;28:e79482. doi: 10.2196/79482 (PMC12978897; doi:10.2196/79482)
Supplement: Multimedia Appendix 3 [file jmir-v28-e79482-s003.docx]

**Appendix methods**

**Introduction**

In this study, our primary goal was prediction, and the secondary goal was risk stratification (i.e., to identify subgroups of patients at similar risk of the outcome). First, we randomly and equally divided beneficiaries into training, testing, and validation samples based on the characteristic and opioid use disorder (OUD) distribution. We developed and tested prediction algorithms for incident OUD using four machine learning approaches: elastic net (EN), random forests (RF), gradient boosting machine (GBM), and least absolute shrinkage and selection operator (LASSO). For each approach, we fit the trained algorithms based on the training sample, refined the algorithm using the testing sample, and then applied the final algorithm in the validation sample to evaluate prediction performance.

Our model reporting complies with the Transparent Reporting of Multivariable Prediction Model for Individual Prognosis or Diagnosis (TRIPOD)+ Artificial Intelligence (AI) reporting guidelines [1]. We calculated the C-statistic (or area under the receiver operating curve [ROC]) from the validation sample to assess discrimination (i.e., the extent to which patients predicted as high-risk exhibit higher OUD rates compared to those predicted as low risk). We examined any difference in C-statistics across different approaches using the DeLong Test [2]. For each probability cutoff point, OUD was predicted for the visits with calculated probabilities above the cutoff point, whereas non-OUD was predicted for the visits with probabilities below the cutoff points. Based on their true and predicted OUD status, the patients’ 3-month visits can be assigned to one of the four groups (i.e., true positive [TP], false positive [FP], true negative [TN], false negative [FN]) shown in the classification matrix (**S3 Fig**). Given that OUD events are rare outcomes and C-statistics do not incorporate information about the prevalence of the outcome, we reported other more appropriate metrics, including sensitivity, specificity, positive predictive value (PPV), negative predictive value (NPV), positive likelihood ratio (PLR), negative likelihood ratio (NLR), number needed to evaluate (NNE) to identify one OUD, and estimated rate of alerts to assess pre-implementation evaluation of our prediction algorithms (**S3 Fig**) [3]. The optimal algorithm for a screening test depends on pre-test probability of the outcome, the values of TPs and TNs, and the costs of FP and FN. Since these factors vary from setting to setting (and some of them are subjective choices), no single cutoff point is suitable for every purpose. In order to compare performance across methods, we presented and assessed these prediction metrics (e.g., NNE) at the optimized threshold of the predicted probability that balances sensitivity and specificity as identified by the Youden index [4], as well as at multiple levels of sensitivity and specificity (e.g., 90%-100%) to allow risk-benefit evaluations of interventions triggered by positive tests using different thresholds defining high risk.

Second, based on the individual’s predicted probability of an OUD event, we classified beneficiaries in the validation sample into decile risk subgroups, with the highest decile further split into three additional strata based on the top 1^st^, 2^nd^ to 5^th^, and 6^th^ to 10^th^ percentiles to allow closer examination of patients at highest risk of developing OUD. We evaluated calibration plots (the extent to which the predicted OUD risk agreed with the observed risks) by the risk subgroup. We briefly summarized our machine learning approaches in the sections below (see details in our previously published work) [5,6].

**Regularized logistic regression: Elastic net (EN)** [7,8]

We chose to use EN regularization because it minimizes overfitting through parameter shrinkage and variable selection to create a parsimonious algorithm. Elastic Net combines both L1 and L2 penalties in its loss function, which helps retain the strengths of both ridge regression and variable selection capabilities. Briefly, after forming a prediction model with logistic regression using all candidate variables, beta coefficients are penalized and lowered to deal with model overfitting. The magnitude of penalization is subsequently changed to create various models with different prediction errors in a cross-validation process, so the final model achieves optimal penalization based on the lowest prediction error. In regularized regression, variable selection is performed automatically by shrinking regression coefficients of some variables to zero. Specifically, the loss function in EN regularization included log likelihood and regularization parameters λ1 and λ2 (smaller values indicating less penalization). The EN procedure generated a total of 40 candidate values for λ1 and λ2 from the training set. We used 5-fold cross-validation and applied the 1-standard error (1-SE) rule to select the optimal λ1 and λ2 in the final model in the training and testing samples. We standardized continuous variables to improve optimization and convergence of the models. Similar to traditional statistical methods (e.g., logistic regression), regularized regression methods cannot handle missing values and delete rows with missing data. For variables with missing information, we imputed with the median for continuous variables and the most frequent category for categorical variables. Our candidate model contained all predictor candidates, quadratic transformation of non-normal distributed continuous variables, and two-way interactions between predictors. Inclusion of quadratic transformations is commonly practiced in conducting EN regularization to accommodate potential non-linear relationships between key candidate predictors and outcomes. Regularized regression is expected to be more effective in the following situations: (1) there are many more columns (predictors) than rows (observations), (2) when the predictors available may be extremely highly correlated with each other, or (3) the goal is to find the most compact model yielding an acceptable performance. We used the sklearn package in Python 3.6 to perform EN. All other parameters were set as default values.

**Tree-structured approaches: Random Forests (RF)** [9,10] **and Gradient Boosting Machine (GBM; Stochastic gradient boosting or TreeNet in Salford SPM)** [11]

This study used two tree ensemble approaches including RF and GBM. An RF consists of a collection of trees grown in parallel, while GBM consists of a series of trees grown in a sequential order of successive trees to minimize residual error. For RF, at each split in a tree, a random sample of predictors is chosen. We followed the steps and rationale from the implementation of Chirkov et al.’s RF framework [12]. Prior to conducting RF, for variables with missing information, we imputed with the median for continuous variables, and with the most frequent category for categorical variables. We used the “*Random Forests Tree Ensembles”* in the software package Salford SPM for this study. The final fine-tuning parameters included the number of trees to build as 200, the number of predictor candidates randomly selected at each node as $\sqrt{number of total predictors}=\sqrt{183}$, using the balanced (i.e., upweight small classes to equal the size of the largest target class) class weight function, and an out of bag (OOB) function while other parameters remained as default. For RF, validation visits were assigned to one of the two predictive categories (i.e., OUD vs. non-OUD) if the probability threshold was >0.62 which was identified from the ROC using the Youden Index.

For GBM, we used the Salford’s TreeNet function to supply an initial value specific to the chosen loss function (i.e. logistic binary) for each record in the training sample. TreeNet can handle missing values automatically. We used cross entropy (i.e., negative average log likelihood) as the tuning criterion to determine the number of trees optimal for logistic models. Second, TreeNet sampled 25% of the records in the training sample randomly and then computed the generalized residual for the records in the sample. The first tree is fitted to the data and begins with a very small tree as the initial model. TreeNet used the sampled records to fit a classification tree with a maximum 8 terminal nodes to the generalized residuals. Third, TreeNet used the classification tree derived from the sampled records to update the TreeNet model based on the loss function and shrink the updated tree by the learning rate (or shrinkage rate) at 0.1 for overfitting protection. TreeNet repeated the steps previously described 200 times (i.e., number of trees to build = 200). Finally, we tested and validated the algorithms in the testing and validation samples. For TreeNet, validation visits were assigned to one of the two predictive categories (i.e., OUD vs. non-OUD) if the probability threshold >0.49 that was identified from the ROC using the Youden Index.

**Regularized logistic regression: Least Absolute Shrinkage and Selection Operator (LASSO)** [13,14]

We employed LASSO regression to develop a parsimonious predictive model for opioid use disorder (OUD). LASSO is a regularization method that applies an L1 penalty to the logistic regression loss function, shrinking some coefficients exactly to zero, thereby performing both variable selection and regularization simultaneously [13]. This approach is particularly effective when the number of candidate predictors is large or when predictors are highly correlated. The LASSO method is advantageous for generating interpretable models with fewer predictors, reducing the risk of overfitting while maintaining predictive accuracy. We implemented LASSO using the sklearn package in Python 3.6, utilizing 5-fold cross-validation in the training set to select the optimal penalty parameter (λ) that minimized prediction error. Continuous predictors were standardized to improve model convergence, and missing data were imputed using the median for continuous and the mode for categorical variables. The final model was evaluated in the testing and validation samples using discrimination and calibration metrics consistent with the other approaches.

**References**

1. Collins GS, Moons KGM, Dhiman P, Riley RD, Beam AL, Van Calster B, Ghassemi M, Liu X, Reitsma JB, Van Smeden M, Others. TRIPOD+ AI statement: updated guidance for reporting clinical prediction models that use regression or machine learning methods. bmj British Medical Journal Publishing Group; 2024;385.

2. DeLong ER, DeLong DM, Clarke-Pearson DL. Comparing the areas under two or more correlated receiver operating characteristic curves: a nonparametric approach. Biometrics JSTOR; 1988;837–845.

3. Romero-Brufau S, Huddleston JM, Escobar GJ, Liebow M. Why the C-statistic is not informative to evaluate early warning scores and what metrics to use. Crit Care 2015;19(1).

4. Fluss R, Faraggi D, Reiser B. Estimation of the Youden Index and its associated cutoff point. Biometrical Journal: Journal of Mathematical Methods in Biosciences 2005;47(4):458–472.

5. Lo-Ciganic W-H, Huang JL, Zhang HH, Weiss JC, Wu Y, Kwoh CK, Donohue JM, Cochran G, Gordon AJ, Malone DC, Others. Evaluation of machine-learning algorithms for predicting opioid overdose risk among medicare beneficiaries with opioid prescriptions. JAMA Netw Open American Medical Association; 2019;2(3):e190968–e190968.

6. Lo-Ciganic WH, Huang JL, Zhang HH, Weiss JC, Kwoh CK, Donohue JM, Gellad. Using machine learning to predict risk of incident opioid use disorder among fee-for-service Medicare beneficiaries: a prognostic study. PLoS One 2020;15(7).

7. Friedman J. The elements of statistical learning: Data mining, inference, and prediction. 2009.

8. Zou H, Hastie T. Regularization and variable selection via the elastic net. J R Stat Soc Series B Stat Methodol Oxford University Press (OUP); 2005;67(2):301–320. doi: 10.1111/j.1467-9868.2005.00503.x

9. Breiman L. Random forests. Mach Learn Springer; 2001;45(1):5–32.

10. Boulesteix A-L, Janitza S, Kruppa J, König IR. Overview of random forest methodology and practical guidance with emphasis on computational biology and bioinformatics. Wiley Interdiscip Rev Data Min Knowl Discov Wiley Online Library; 2012;2(6):493–507.

11. Friedman JH. Greedy function approximation: a gradient boosting machine. Ann Stat JSTOR; 2001;1189–1232.

12. Chirikov V V, Shaya FT, Onukwugha E, Mullins CD, Howell CD, Others. Tree-based claims algorithm for measuring pretreatment quality of care in Medicare disabled hepatitis C patients. Med Care LWW; 2017;55(12):e104–e112.

13. Tibshirani R. Regression shrinkage and selection via the lasso. J R Stat Soc Series B Stat Methodol Oxford University Press; 1996;58(1):267–288.

14. Jonas R, Cook J. LASSO regression. British Journal of Surgery John Wiley & Sons, Inc.; 2018;105(10).
